# Biochemical aspects of seeds from *Cannabis sativa* L. plants grown in a mountain environment

**DOI:** 10.1038/s41598-021-83290-1

**Published:** 2021-02-16

**Authors:** Chiara Cattaneo, Annalisa Givonetti, Valeria Leoni, Nicoletta Guerrieri, Marcello Manfredi, Annamaria Giorgi, Maria Cavaletto

**Affiliations:** 1 Dipartimento di Scienze e Innovazione Tecnologica-DiSIT, Università del Piemonte Orientale, 13100 Vercelli, Italy; 2 Centre of Applied Studies for the Sustainable Management and Protection of Mountain Areas (CRC Ge.S.Di.Mont.), University of Milan, Via Morino 8, 25048 Edolo, (BS) Italy; 3 Department of Agricultural and Environmental Sciences - Production, Landscape, Agroenergy (DISAA), Via Celoria 2, 20133 Milan, Italy; 4 Water Research Institute-National Research Council (IRSA-CNR), Verbania, Italy; 5 Centro di Ricerca Traslazionale sulle Malattie Autoimmuni e Allergiche– CAAD, Università del Piemonte Orientale, 28100 Novara, Italy

**Keywords:** Biochemistry, Plant sciences

## Abstract

*Cannabis sativa* L. (hemp) is a versatile plant which can adapt to various environmental conditions. Hempseeds provide high quality lipids, mainly represented by polyunsaturated acids, and highly digestible proteins rich of essential aminoacids. Hempseed composition can vary according to plant genotype, but other factors such as agronomic and climatic conditions can affect the presence of nutraceutic compounds. In this research, seeds from two cultivars of *C. sativa* (Futura 75 and Finola) grown in a mountain environment of the Italian Alps were analyzed. The main purpose of this study was to investigate changes in the protein profile of seeds obtained from such environments, using two methods (sequential and total proteins) for protein extraction and two analytical approaches SDS-PAGE and 2D-gel electrophoresis, followed by protein identification by mass spectrometry. The fatty acids profile and carotenoids content were also analysed. Mountain environments mainly affected fatty acid and protein profiles of Finola seeds. These changes were not predictable by the sole comparison of certified seeds from Futura 75 and Finola cultivars. The fatty acid profile confirmed a high PUFA content in both cultivars from mountain area, while protein analysis revealed a decrease in the protein content of Finola seeds from the experimental fields.

## Introduction

*Cannabis sativa* L. (hemp) is an annual plant belonging to the family of *Cannabaceae*. *C. sativa* is naturally dioecious, however this plant has been domesticated by humans since the prehistoric era and monoecious varieties have been selected to obtain higher quality fibers and to optimize seed harvest procedures^1^. Hemp versatility favoured its spreading worldwide: in fact it has been used for a long time for the production of fibers, paper, oils and medicaments^2^. Hemp industry declined at the beginning of the 20th Century, when cannabis was banned due to the discovery of the psychoactive molecule Δ-9-tetrahydrocannabinol (THC), which content is high in some hemp varieties. Besides THC, *Cannabis sativa* produces a plethora of phytocannabinoids, such as cannabidiolic acid (CBDA), which is converted in CBD and does not exert psychoactivity. These molecules are produced and stored in the glandular trichomes of the inflorescence, especially of the female plant^3^. Hemp varieties can be discriminated in different chemotypes, depending on the amount and ratio of CBD and THC: Chemotype I presents high THC (> 0.3% d.w. female inflorescence) and low CBD content; Chemotype II is an intermediate type with the presence of similar amounts of THC and CBD; Chemotype III shows high CBD and low THC amount (less than 0.3%). The latter type is known as “fiber type” and includes the varieties of *C. sativa* allowed for industrial purposes^4^. At the end of the 90 s a renewed interest in hemp has been triggered by its high plasticity of growth and by the multipurpose applicability of its products. Beyond fiber and paper production, hemp is a suitable plant for the restoration of contaminated soils, and its biomass can be used for the production of biomaterials and biofuels such as bioethanol^5^. The interest in hemp industry can be argued by the number of hemp cultivars registered in the European Plant Variety Database, raising from 12 in 1995^6^ to the current 75 varieties allowed for industrial cultivation (European Commission EU Plant Variety Database), with an increasing effort in breeding plants for multi-purpose production (i.e. seeds and fibers). Hempseeds, which for a long time have been considered like “by products” of hemp cultivation and used as animal feed, have been reconsidered for their content of high quality oils and proteins^7^. Hemp shows high adaptability to various conditions, however environmental factors such as climatic conditions and elevation may influence not only the plant growth but also the nutritional and organoleptic traits of seeds^8^, therefore it is important to choose a variety (cultivar) optimized for the specific application and also adapted to the environment of growth. There is scarce information regarding hemp growth in mountain environments, however it has been observed that the elevation could affect the content of relevant molecules such as secondary metabolites and fatty acids^9^. Hemp seeds are rich in polyunsaturated fatty acids (PUFA), mainly linoleic acid (LA, omega-6) and α-linolenic acid (ALA, omega-3), which cannot be synthesized by mammals and must be assumed with the diet. Hemp seeds contain an optimal omega-6/omega-3 ratio for human nutrition, which has been defined as 3:1. The most abundant proteins of seeds are represented by storage proteins, which are localized in protein bodies of mature seeds, providing a source of amino acids during germination and plant development. Seed storage proteins are a mixture of isoforms encoded by multigene families^10^. The classification of storage proteins is based on the Osborne fractionation and sequential extraction^11^, where seed proteins were clustered on the basis of solubility in water (albumins), dilute saline (globulins), alcohol/water mixtures (prolamins) and dilute alkali or acid (glutelins). However, each fraction is a complex mixture of different proteins sharing a similar solubility. Albumin and globulin fractions include enzymes involved in metabolic activities but also many uncharacterized proteins^12^. Storage globulins (11S legumin-like) are located in the endosperm and are composed by subunits of 50 kDa, which are post-translationally cleaved to obtain acid (30 kDa) and basic (20–22 kDa) chains, linked by a disulfide bond^13^. In this research, seeds from two cultivars of *C. sativa*, the monoecious Futura 75 and the dioecious Finola, grown in mountain environments of Verbano Cusio Ossola (Italy) during the growing season 2018 were analyzed. The main purpose of this study was to investigate changes in the protein profile of seeds obtained from a mountain environment, using different methods of protein extraction (sequential and total proteins) and separation (SDS-PAGE and 2D-gel electrophoresis) to gain a more detailed information of the fractions that could be affected. The fatty acids profile and carotenoids content were also analysed, to gain a global view of nutraceutical substances contained in hempseeds.

## Results and discussion

### Growing conditions and seed weight

Weather conditions are summarized in Figure S1. During the growing season (May–September) the lowest temperatures in the Crodo area were registered in May and September (15–18 °C) and the maximum in the months of June and July (22 °C). Concerning rainfall, the highest value was detected in May (192 mm), precipitations were stable from June to August (70–80 mm), and the lowest value was detected in September (23 mm). Mountain environments can show prompt changes among valleys. The minimum and maximum temperatures registered in Viganella area were 9 °C in May and 17 °C in July–August, respectively. The rainfall was much higher respect with Crodo, with 306 mm registered in May, 97–150 mm during the June–August period, and a final decrease in September (60 mm).

The seeds of Futura 75 and Finola varieties that were used to set up the experimental fields were included in the analyses and considered as reference standards (named “certified seeds”) to be compared with the seeds of Futura 75 and Finola that were obtained from the experimental fields (harvested seeds). The weight distribution of 50 seeds from each variety and experimental field is shown in the box-plot of Fig. S2. Futura 75 seeds show a higher weight respect with Finola seeds, irrespective of their origin (harvested or certified seeds). Harvested seeds of Futura 75 were smaller than the certified seeds, but the weight of harvested seeds was similar in both the experimental fields (Table 1). Concerning Finola variety, the weight of certified and harvested seeds from the Crodo field was similar, while a weight decrease was observed for harvested seeds from Viganella respect with the Crodo field.

**Table 1:** Mean weight (g) and standard deviation (SD) of a representing sample of 50 seeds from Finola and Futura 75 varieties, obtained from the experimental fields of Viganella and Crodo. Certified seeds are included as a reference. The last column reports the percentage of weight difference of harvested seeds respect with certified seeds of the same hemp variety.

Variety	Source	Mean	SD	% Difference versus certified seeds (%)
Futura 75	Certified	0.923	0.008	
Futura 75	Viganella	0.779	0.027	− 15.6
Futura 75	Crodo	0.788	0.021	− 14.59
Finola	Certified	0.617	0.017	
Finola	Viganella	0.475	0.054	− 23.1
Finola	Crodo	0.600	0.024	− 2.8

The protein yield of certified seeds resulted in 64.4 ± 5.1 mg/ml for Futura 75 and 62.6 ± 6.1 mg/ml for Finola, for a total protein content of 29 and 34.4 mg starting from 1 g of seeds. Regarding the experimental field of Crodo, the protein yield was 53.6 ± 5.3 mg/ml for Futura 75 and 56.2 ± 5.3 mg/ml for Finola, for a total protein content of 37.5 and 30.9 mg starting from 1 g of seeds. Finally, the protein yield of Viganella seeds was 61.2 ± 3.5 mg/ml for Futura 75 and 34.9 ± 7.2 mg/ml for Finola, with a total protein content of 23.3 and 19.2 mg starting from 1 g of seeds.

Protein content was more similar between varieties from the same experimental field rather than considering the same variety between different experimental fields, suggesting a climatic effect on the plant response. The higher level of precipitations and the lower temperatures detected in Viganella respect with Crodo during the growing period could have negatively affected protein content of seeds from Viganella. These observations are in agreement with other experimental evidencies. The comparison of hemp seeds obtained from three different growing years revealed a higher protein content in seeds from the year with the lowest rainfall^8^ and in soybean, the protein content of seeds was found to be positively correlated with temperature and negatively correlated with rainfall during seed development^14^.

### Photosynthetic pigments

The total content of carotenoids observed for both varieties was similar (4 mg/100 g) in certified and Viganella seeds; these values are in line with those reported in^8,15^, whereas a higher amount was observed for Finola seeds of Crodo (Fig S3). The carotenoid/chlorophyll ratio of Finola seeds was lower compared with Futura 75, except for seeds from Viganella, which showed the highest ratio together with Futura 75 seeds from Crodo (0.66 and 0.68 respectively).

Currently there are few studies regarding carotenoids content of hempseeds, with the majority of them referring to the oil, however variations due to both genotype and year of cultivation were reported^8^. Carotenoids are part of the unsaponifiable fraction of hempseed oil, together with tocopherols and phytosterols, and their antioxidant activity contribute to preserve the oxidative stability of polyunsaturated fatty acids. Lutein was found to be the most abundant carotenoid in Finola, Futura and other five hemp varieties compared by^8^ and total carotenoid content can vary depending on both genotype and climatic conditions.

Considering that residual chlorophylls in seed reduce seed tolerance against stressful conditions, and the increase of the ratio of carotenoid to chlorophyll content is a measure of seed tolerance to stress^16^, we can suggest that Finola seeds from Crodo are less resistant than Finola seeds from Viganella.

### Seed fatty acid profile

The results of fatty acid profiling of Finola and Futura 75 seeds and the total of saturated fatty acids (SFA), monounsaturated (MUFA), polyunsaturated (PUFA), omega-3 (ω3) and omega-6 (ω6) fatty acids are shown in Table 2.

**Table 2:** Fatty acid profile and composition (w/w%) in Finola and Futura 75 seeds. Fatty acid contents are expressed as means ± SD (n = 3, independent biological replicates). Significant different values (P < 0.05, Tukey post-hoc test, Bonferroni adjustment) are indicated by different letters.

		Futura75 Viganella		Futura75 Crodo		Futura75 Cert		Finola Viganella		Finola Crodo		Finola Cert	
Fatty acid	Abbreviation	Mean	SD	Mean	SD	Mean	SD	Mean	SD	Mean	SD	Mean	SD
Miristic	C14:0	0.05^b^	0.01	0.06^b^	0.01	0.00^b^	0.00	0.03^b^	0.00	0.04^b^	0.01	0.33^a^	0.15
Pentadecanoic	C15:0	0.02	0.01	0.02	0.01	0.00	0.00	0.02	0.01	0.02	0.01	0.30	0.20
Palmitic	C16:0	7.21^ab^	0.25	7.26^ab^	0.06	7.00^a^	0.00	6.81^a^	0.03	7.62^b^	0.09	7.13^ab^	0.36
Palmitoleic	C16:1	0.13	0.00	0.14	0.01	0.10	0.00	0.10	0.01	0.12	0.00	0.36	0.47
Eptadecanoic	C17:0	0.06	0.01	0.06	0.00	0.00	0.00	0.05	0.00	0.05	0.00	0.24	0.40
Cis-10 eptadecanoic	C17:1	0.00	0.00	0.00	0.00	0.00	0.00	0.00	0.00	0.00	0.00	0.08	0.10
Stearic	C18:0	3.20	0.03	2.78	0.04	3.40	0.40	3.52	0.02	2.71	0.01	3.09	0.65
Elaidic	C18:1n9t	0.00	0.00	0.00	0.00	0.00	0.00	0.00	0.00	0.00	0.00	0.02	0.02
Oleic	C18:1n9c	13.53^b^	0.09	11.38^c^	0.05	12.60^c^	0.40	13.88^b^	0.14	12.51^c^	0.17	8.82^a^	0.92
Linoleic	C18:2n6c	55.86	0.14	55.38	0.26	56.10	0.20	56.18	0.03	57.34	0.24	56.04	2.41
Arachic	C20:0	0.83	0.04	0.99	0.04	0.90	0.10	0.86	0.03	0.84	0.02	0.78	0.10
γ-Linolenic	C18:3n6	2.15^bc^	0.06	4.41^a^	0.20	2.50^c^	0.40	1.89^b^	0.05	2.36^bc^	0.03	4.33^a^	0.24
Cis-11-eicosenoic	C20:1	0.37^b^	0.01	0.42^b^	0.00	0.30^b^	0.00	7.64^a^	0.99	7.65^a^	2.39	0.35^b^	0.18
Linolenic	C18:3n3	15.97^b^	0.10	16.24^b^	0.01	16.40^b^	0.50	8.76^a^	0.98	8.57^a^	2.33	15.88^b^	0.81
Heneicosanoic	C21:0	0.02	0.01	0.02	0.00	0.00	0.00	0.02	0.01	0.00	0.00	0.07	0.12
Cis-11,14-eicosadienoic	C20:2	0.02^b^	0.02	0.05^b^	0.01	0.00^b^	0.00	0.04^b^	0.01	0.06^b^	0.01	1.25^a^	0.08
Behenic	C22:0	0.35^a^	0.02	0.45^b^	0.02	0.50^b^	0.30	0.11^a^	0.17	0.00^a^	0.01	0.27^a^	0.05
Cis-8,11,14-eicosatrienoic	C20:3n6	0.00	0.00	0.01	0.00	0.00	0.00	0.01	0.01	0.01	0.01	0.00	0.00
Erucic	C22:1n9	0.02	0.01	0.03	0.01	0.00	0.00	0.02	0.00	0.03	0.00	0.40	0.10
Cis-11,14,17-eicosatrienoic	C20:3n3	0.00	0.00	0.01	0.00	0.00	0.00	0.00	0.00	0.00	0.00	0.00	0.00
Arachidonic	C20:4n6	0.03^a^	0.01	0.06^a^	0.01	0.00^b^	0.00	0.00^b^	0.00	0.00^b^	0.00	0.00^b^	0.00
Cis-13,16- docosadienoic	C22:2	0.02	0.01	0.00	0.00	0.00	0.00	0.00	0.00	0.00	0.00	0.04	0.02
Lignoceric	C24:0	0.15	0.01	0.23	0.01	0.10	0.10	0.04	0.00	0.00	0.00	0.14	0.08
Cis-5,8,11,14,17-eicosapentaenoic	C20:5n3	0.00	0.00	0.00	0.00	0.00	0.00	0.05	0.06	0.02	0.01	0.04	0.02
Nervonic	C24:1	0.01^b^	0.00	0.02^b^	0.01	0.00^b^	0.00	0.00^b^	0.00	0.00^b^	0.00	0.04^a^	0.01
Saturated Fatty Acids (SFA)		11.89	0.19	11.86	0.07	11.93	0.35	11.41	0.16	11.29	0.12	12.35	0.91
Monounsaturated Fatty Acids (MUFA)		14.06^a^	0.09	11.98^a^	0.03	13.00^a^	0.36	21.65^b^	1.12	20.35^b^	2.39	10.08^a^	1.61
Polyunsaturated Fatty Acids (PUFA)		74.05^a^	0.10	76.16^a^	0.09	75.07^a^	0.61	66.94^b^	0.98	68.36^b^	2.27	77.58^a^	2.48
Omega-3 (ω-3)		15.97^a^	0.10	16.25^a^	0.01	16.39^a^	0.54	8.82^b^	1.03	8.59^b^	2.33	15.93^a^	0.80
Omega-6 (ω-6)		58.08	0.10	59.90	0.09	58.65	0.25	58.12	0.06	59.77	0.23	60.40	2.28
ω-6/ω-3		3.64		3.69		3.58		6.59		6.96		3.80	

The principal SFA was palmitic acid (PA; 16:0) for Futura 75 (Viganella 7.21- Crodo 7.26%) and Finola (6.81–7.62%), followed by stearic acid (SA; 18:0) values of 3.20–2.78% in Futura 75, and 3.52- 2.71% in Finola seeds from the fields of Viganella and Crodo respectively. The total SFA content was similar in the two varieties (11.89–11.86% in Futura 75, and 11.41–11.29% in Finola grown in Viganella and Crodo). The most abundant unsaturated fatty acids in the seeds of the two varieties were linoleic acid (LA; C18:2 ω6c): 55.86–55.38% for Futura 75 and 56.18–57.34% in Finola grown in Viganella and Crodo, and oleic acid (OA; C18:1 ω9c): 13.53–11.38% for Futura 75 and 13.88–12.51% for Finola grown in Viganella and Crodo, respectively, with a lower value observed in certified seeds of Finola (8.82%). Similar amounts of ω3 linolenic acid (C18:3ω3) were found in certified seeds of both Futura 75 and Finola (16.4–15.88%) and Futura 75 seeds obtained from the two experimental fields (15.97–16.24%). Lower amounts were detected in Finola seeds from both Viganella and Crodo (8.76–8.57%). A significant amount of Cis-11-eicosenoic acid (C20:1) was found in Finola from the two experimental fields (7.64–7.65%), while low amounts (below 0.5%) were present in both certified and harvested seeds of Futura 75 and certified Finola seeds. According to this, the total MUFA percentage resulted higher in harvested seeds of Finola (21.65–20.35%) respect with harvested Futura 75 (14.06–11.98%) and certified seeds of both varieties (13–10.08%). PUFA represent the main source of fatty acids in the seeds of both varieties. A comparable amount was observed in certified (75.07%) and harvested seeds of Futura 75 (74.05–76.16%), while a slightly lower amount was detected in harvested (66.94–68.36%) compared with certified (77.58%) Finola seeds. Both the genotypes are rich in the two essential fatty acids (EFAs) LA (18:2 ω6) and α-linolenic acid (18:3 ω3). Futura 75 seeds from Crodo showed the highest content of γ-linolenic (GLA, 4.41%), which is comparable with the value observed in certified seeds of Finola (4.33%), while harvested Finola seeds presented a linolenic acid content two times lower than Futura 75, with consequently higher average values of ω6/ω3 ratio (6.59–6.96 Finola vs. 3.64–3.69 harvested Futura 75, and 3.58–3.80 certified seeds of Futura 75 and Finola). These observations are in accordance with^9^ where a decrease of ω3 linolenic acid and increase of Cis-11-eicosenoic acid were observed in seeds of Finola respect with Futura 75 plants grown in mountain environment of Italian Alps, with a similar ω6/ω3 ratio in Finola seeds (6.25).

Hempseeds represent a rich source of high quality fatty acids (representing the 25–35% of the seed) whose composition can vary among different cultivars, with possible adaptive significance. Both Futura 75 and Finola varieties grown in mountain fields have been shown as a good source of PUFA. According to the European Food and Safety Authority (EFSA) the ideal ω6/ω3 ratio is in the range 3:1–5:1 and the values obtained from hempseed oil of different cultivars fall within this range^4^. The ω6/ω3 ratios obtained for certified seeds of Finola and both certified and harvested seeds of Futura 75 are consistent with those reported in literature^17^, while harvested seeds of Finola contained lower amount of ω3 linolenic acid. The other significant variation observed in harvested Finola seeds regards the monounsaturated Cis-11-eicosenoic acid (C20:1, EA). EA presents interesting properties and is used as a raw material in the production of cosmetics, lubricant oils and pharmaceutical compounds. EA is a precursor of erucic acid in higher plants and the production of erucic acid is usually higher than EA. Its synthesis is supposed to be performed by D9-fatty acid elongase (D9EL) from oleic acid. Notably, a temperature dependent increase in the production of EA was observed in PUFA producer fungi belonging to *Mortierella* species, grown below 20°C^18^. Lysophosphatidylcholine acyltransferase 2 (LPCAT2) was found to be the principal determinant of variation in C20:1 content of *A. thaliana* seeds. Reduced expression of LPCAT2 is correlated with the enhanced production of EA at the expense of PUFA, shifting the fate of the acyl-CoA pool from the incorporation into phosphatidylcoline to the fatty acid elongation^19^.

The observed increase of MUFA in Finola seeds from the experimental fields suggests an adaptive plasticity to the environment of growth. It is widely demonstrated that fatty acid composition is related to temperature, which shows a positive correlation with saturated fatty acids content, while generally an increase of unsaturated acids is observed in plants growing at lower temperatures. In this study, high levels of linoleic acids were observed in all the analyzed seeds without statistical differences among the samples. A strong correlation between fatty acids composition and environmental factors such as elevation, maximum temperature and precipitation was observed in seeds of *Sapindus* spp., where EA specifically correlated with elevation^20^. The lower linolenic acid and the higher EA content of harvested Finola respect with certified Finola and both harvested and certified Futura 75 seeds, confirm previous observations from another Italian Alpine environment^9^ possibly refecting an adaptation of Finola plants to this environment.

### Protein analysis of sequential fractions

**Figure 1:**
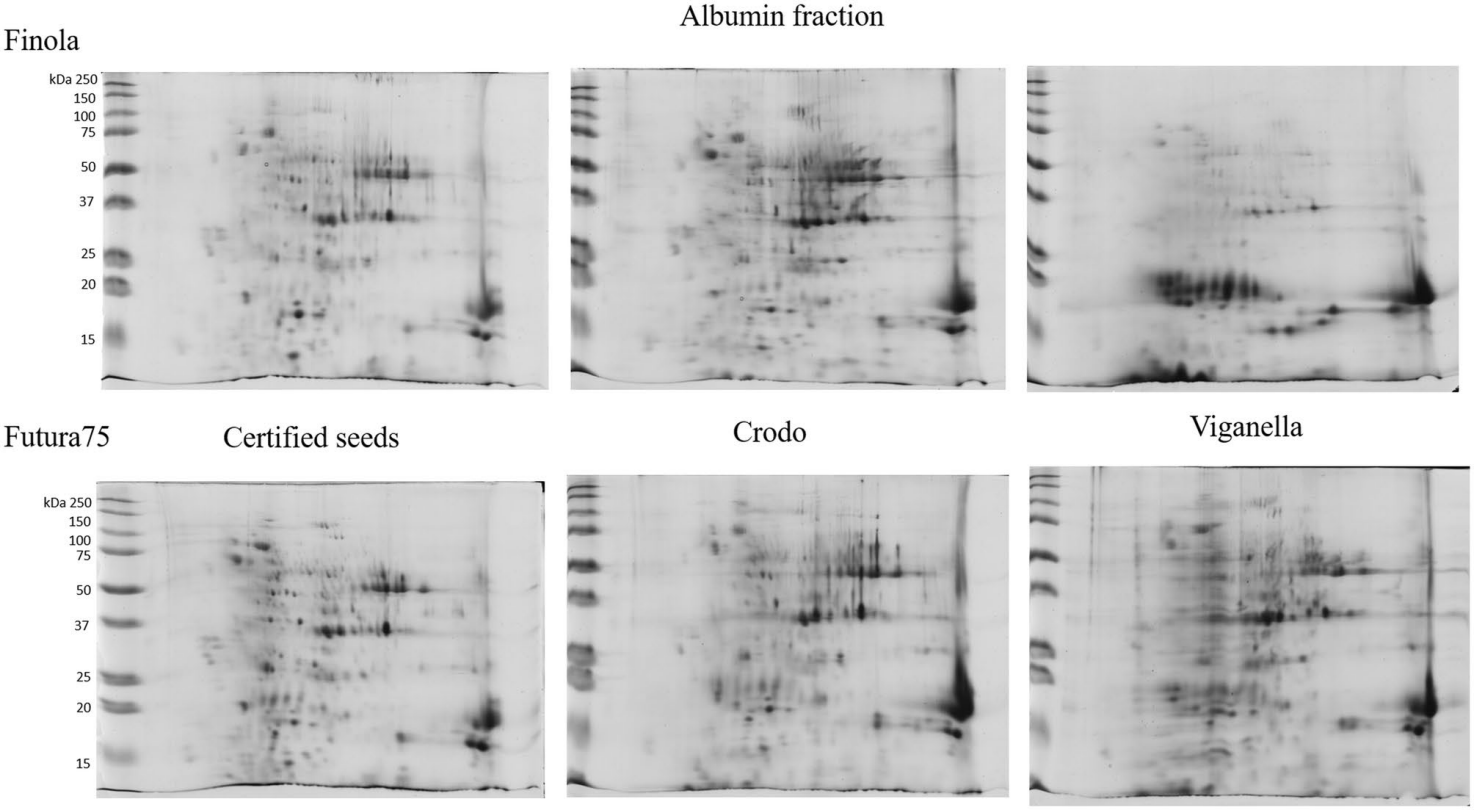
2D-PAGE of albumin fraction of Finola (on the upper part of the image) and Futura 75 (on the lower part of the image) certified seeds (on the left), or obtained from the experimental fields of Crodo (middle) and Viganella (right).

**Figure 2:**
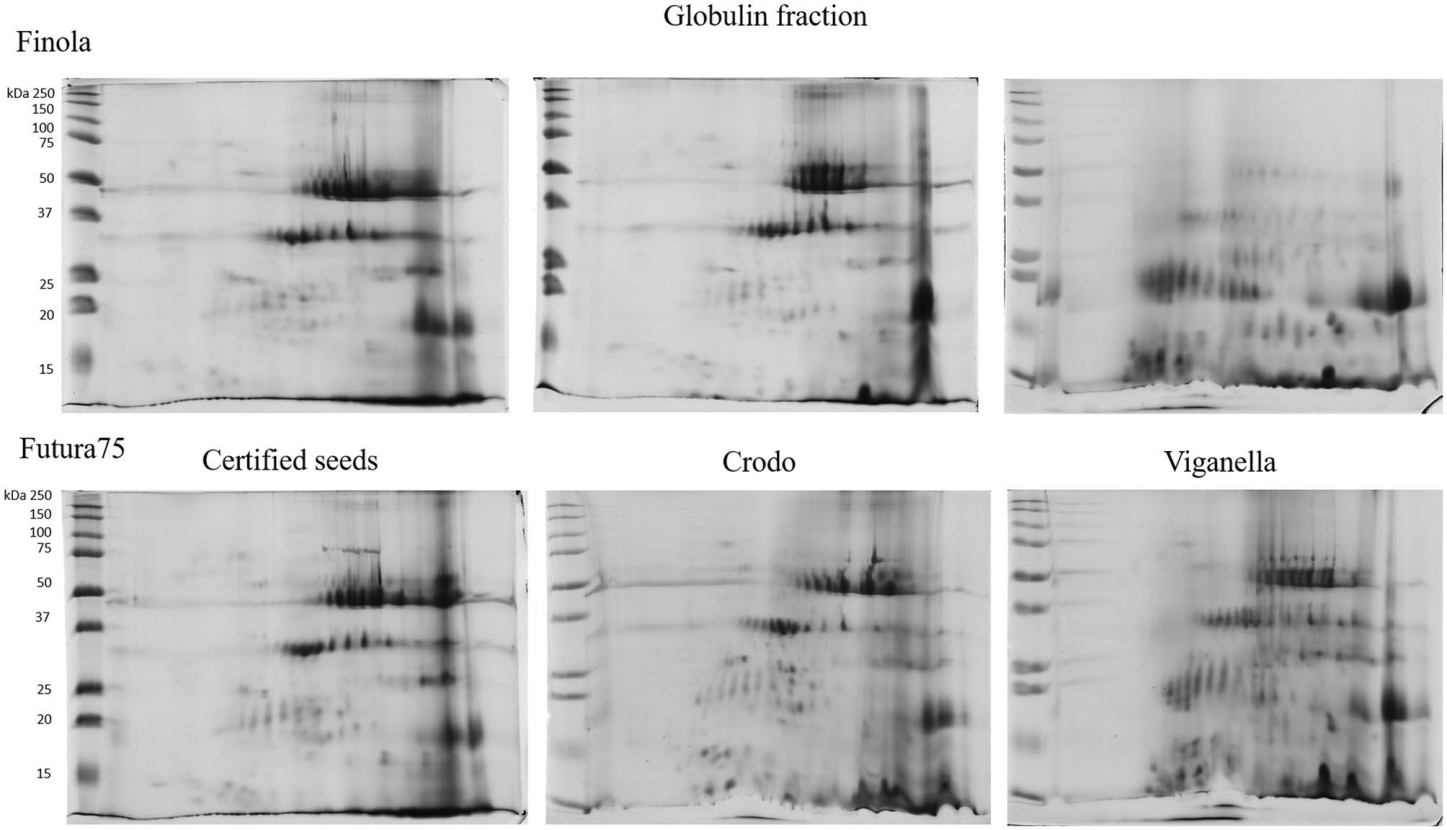
2D-PAGE of globulin fraction of Finola (on the upper part of the image) and Futura 75 (on the lower part of the image) certified seeds (on the left), or obtained from the experimental fields of Crodo (middle) and Viganella (right).

**Figure 3:**
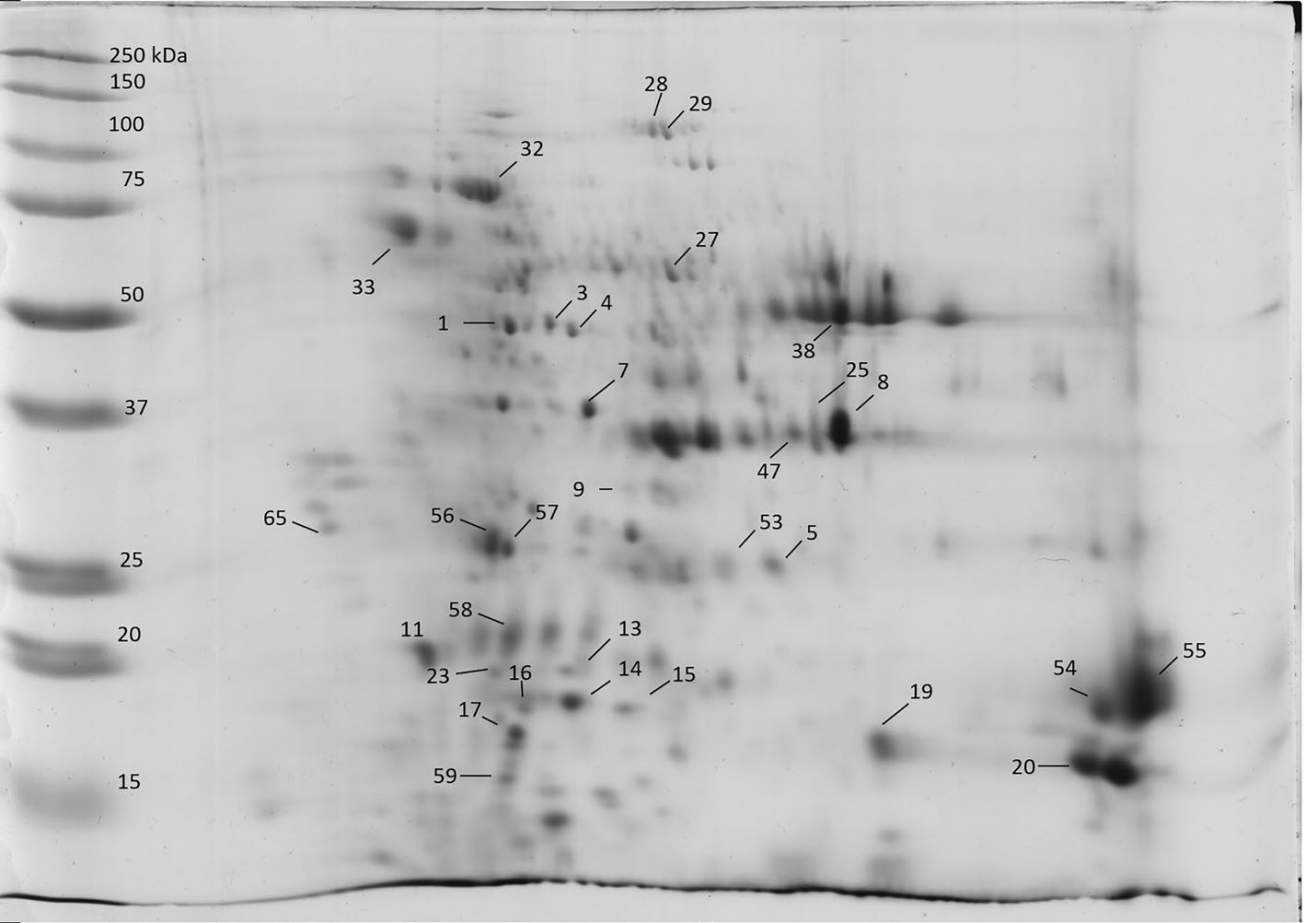
Representative 2D gel of the albumin fraction: the spots identified by MS analysis are numbered.

**Figure 4:**
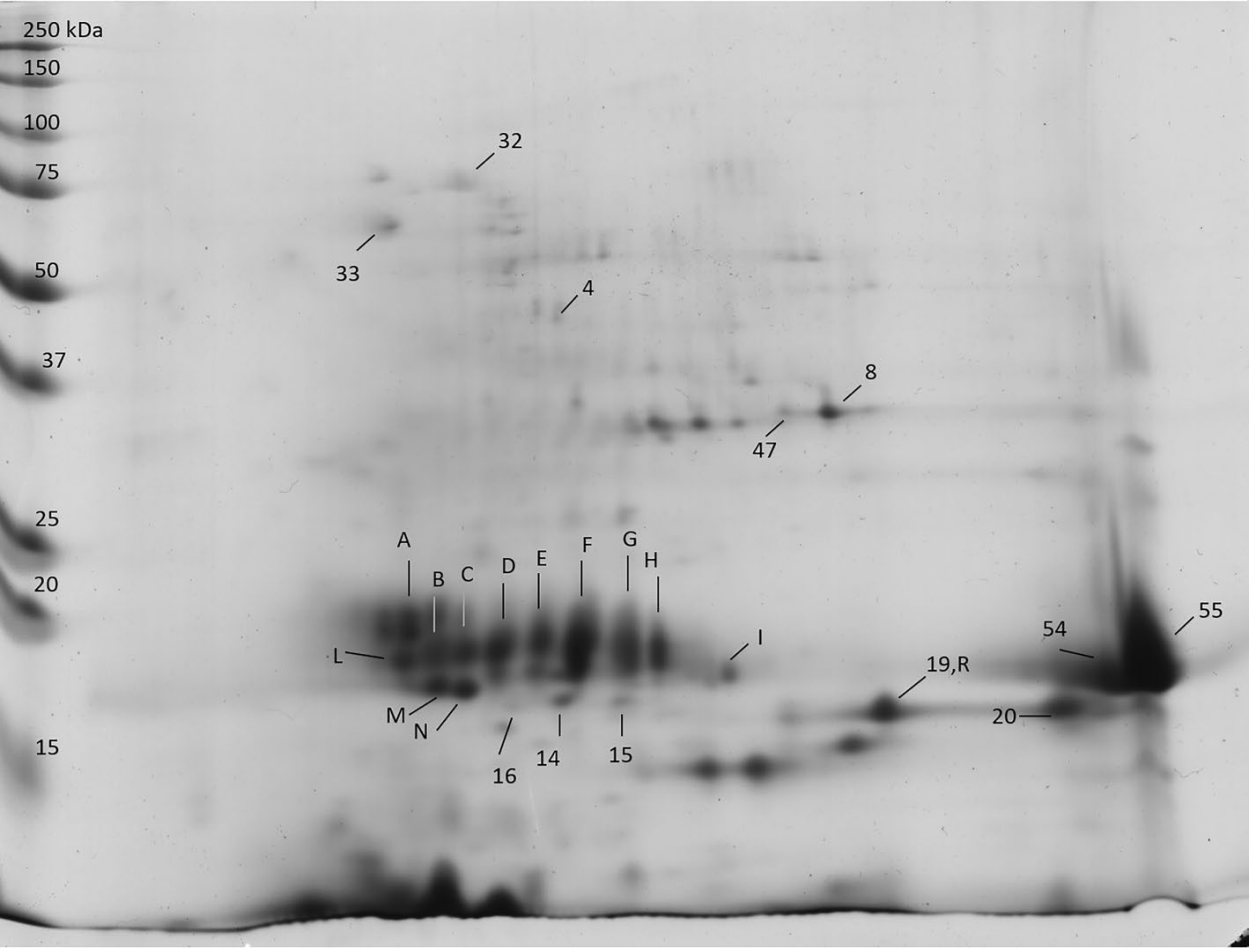
2D gel of the albumin fraction from samples of Finola grown in Viganella: the spots identified by MS analysis are indicated by numbers and letters.

The sequential fractions and total protein extracts from seeds of Finola and Futura 75 cultivars grown in Crodo and Viganella experimental areas, together with certified seeds as a control, were analysed combining both SDS-PAGE and 2D-PAGE, in order to quickly visualize main differences in protein expression among cultivars and experimental fields, and to better characterize the distribution of hempseed proteins. The protein pattern of albumin and globulin fractions was similar in all the samples, except for those obtained from Finola seeds of Viganella, where a dramatic change due to the appearance of several spots at low molecular weight was observed (Figs. 1 and 2 and supplementary figures S4, S5). Concerning the glutelin-like fractions, no differences were observed among the samples (Fig. S6). The albumin fraction contains the highest heterogeneity of proteins and in attempt to identify their function, spots from this fraction were excised from the gels (Figs. 3 and 4) and analyzed by MS. The list of proteins that were identified from albumin fraction is indicated in Table 3 and the peptide sequences are available in supplementary table S1.

**Table 3:** Proteins identified after MS/MS analysis of albumin protein extracts from hemp seeds of Futura75 and Finola varieties analyzed by 2D-PAGE. Spot number, database accession number, protein name and organism are indicated.

Spot	Accession number	Protein name
1	OAP03364.1	SRG3 (*Arabidopsis thaliana*)
3	XP_021757355.1	Phosphoglycerate kinase 3, cytosolic (*Chenopodium quinoa*)
	GER33867.1	Phosphoglycerate kinase (*Striga asiatica*)
	KAF4347106.1	Hypothetical protein G4B88_025149 (*Cannabis sativa*)
	XP_030508778.1	ADP-ribosylation factor 1 (*Cannabis sativa*)
4	XP_030485298.1	Hexokinase-2, chloroplastic (*Cannabis sativa*)
5	XP_030507162.1	1-Cys peroxiredoxin (*Cannabis sativa*)
7	LGUL_ORYSJ	Lactoylglutathione lyase (*Oryza sativa subsp. japonica*)
8	CDP79027.1	Edestin 2 (*Cannabis sativa*)
	XP_030506286.1	NADPH-dependent aldehyde reductase 1, chloroplastic-like (*Cannabis sativa*)
11	RVX02887.1	Putative ribonuclease H protein (*Vitis vinifera*)
13	XP_030504301.1	18.5 kDa class I heat shock protein (*Cannabis sativa*)
	XP_030506109.1	18.5 kDa class I heat shock protein-like (*Cannabis sativa*)
14	XP_030503293.1	18.5 kDa class I heat shock protein-like (*Cannabis sativa*)
15	XP_030485488.1 XP_030488139.1	18.1 kDa class I heat shock protein-like (*Cannabis sativa*)
16	XP_030503293.1	18.5 kDa class I heat shock protein-like (*Cannabis sativa*)
	TKS00882.1	NADH dehydrogenase (*Populus alba*)
17	XP_030499617.1	Glycine-rich RNA-binding protein-like (*Cannabis sativa*)
19	CDP79023.1	Edestin 1 (*Cannabis sativa*)
20	KAF4398011.1 XP_030491271.1	Hypothetical protein G4B88_019732 (*Cannabis sativa*), Thioredoxin-like protein CXXS1 (*Cannabis sativa*)
23	KAF4398011.1 XP_030491271.1	Hypothetical protein G4B88_019732 (*Cannabis sativa*), Thioredoxin-like protein CXXS1 (*Cannabis sativa*)
25	XP_030506286.1	NADPH-dependent aldehyde reductase 1, chloroplastic-like (*Cannabis sativa*)
27	CAB00000.1	Ribulose-1,5-bisphosphate carboxylase/oxygenase large subunit, partial (chloroplast) (*Caryocar glabrum*)
	CDP79023.1	Edestin 1 (*Cannabis sativa*)
28	KAF4398011.1 XP_030491271.1	Hypothetical protein G4B88_019732 (*Cannabis sativa*), Thioredoxin-like protein CXXS1 (*Cannabis sativa*)
29	ABX09991.1	Actin 1, partial (*Ziziphus jujuba*)
	CDP79023.1	Edestin 1 (*Cannabis sativa*)
	PNY00005.1	Tubulin alpha-3 chain-like protein (*Trifolium pratense*)
	AGJ50594.1	Beta-tubulin (*Pericallis cruenta*)
	XP_023875501.1	GTP-binding protein rhoA (*Quercus suber*)
32	XP_030478962.1	Heat shock 70 kDa protein-like (*Cannabis sativa*)
	XP_030501851.1 XP_030492979.1	Luminal-binding protein 5 isoform X1 (*Cannabis sativa*) and mediator of RNA polymerase II transcription subunit (*Cannabis sativa*)
33	RUBA_RICCO	RuBisCO large subunit-binding protein subunit alpha (Fragment) (*Ricinus communis)*
	PDI11_ARATH	Protein disulfide isomerase-like 1–1 (*Arabidopsis thaliana*)
38	XP_030508280.1 XP_030508281.1	Vicilin C72-like (*Cannabis sativa*)
47	HSP11_DAUCA	17.8 kDa class I heat shock protein (*Daucus carota*)
53	XP_030507162.1	1-Cys peroxiredoxin (*Cannabis sativa*)
	XP_030486208.1	Glutathione S-transferase DHAR2-like (*Cannabis sativa*)
54	XP_030482081.1	11 kDa late embryogenesis abundant protein (*Cannabis sativa)*
	SNQ45158.1	Edestin 3 (*Cannabis sativa*)
	SNQ45160.1	Edestin 3 (*Cannabis sativa*)
	CDP79023.1	Edestin 1 (*Cannabis sativa*)
	CP19D_ARATH	Peptidyl-prolyl cis–trans isomerase CYP19-4 (*Arabidopsis thaliana*)
55	SNQ45160.1	Edestin 3 (*Cannabis sativa*)
	SNQ45158.1	Edestin 3 (*Cannabis sativa*)
	CDP79023.1	Edestin 1 (*Cannabis sativa*)
	CDP79028.1	Edestin 2 (*Cannabis sativa*)
	XP_030494449.1	60S ribosomal protein L12-3-like (*Cannabis sativa*)
56	SNQ45153.2 XP_030504501.1	7S vicilin-like protein (*Cannabis sativa*)
	XP_030487452.1	Triosephosphate isomerase, chloroplastic (*Cannabis sativa*)
57	SNQ45153.2 XP_030504501.1	7S vicilin-like protein (*Cannabis sativa*)
58	CDP79023.1, CDP79024.1, CDP79026.1	Edestin 1 (*Cannabis sativa*)
59	XP_030479619.1	Late embryogenesis abundant protein, group 3-like (*Cannabis sativa)*
65	OVA13829.1	Ubiquitin-associated domain/translation elongation factor EF-Ts (*Macleaya cordata*)
A	CDP79023.1, CDP79024.1, CDP79026.1	Edestin 1 (*Cannabis sativa*)
B	CDP79023.1, CDP79024.1, CDP79026.1	Edestin 1 (*Cannabis sativa*)
C	CDP79023.1, CDP79024.1, CDP79026.1	Edestin 1 (*Cannabis sativa*)
C	OAP03364.1	SRG3 (*Arabidopsis thaliana*)
D	CDP79023.1, CDP79024.1, CDP79026.1	Edestin 1 (*Cannabis sativa*)
E	CDP79023.1, CDP79024.1, CDP79026.1	Edestin 1 (*Cannabis sativa*)
F	SNQ45158.1	Edestin 3 (*Cannabis sativa*)
F	CDP79023.1, CDP79024.1, CDP79026.1	Edestin 1 (*Cannabis sativa*)
F	VAH03463.1 (HSP7F_ARATH)	Unnamed protein product (*Triticum turgidum* subsp. Durum) (Heat shock 70 kDa protein 6, chloroplastic (*Arabidopsis thaliana*))
F	XP_004290029.1	PREDICTED: chaperonin CPN60-2, mitochondrial-like (*Fragaria vesca* subsp. Vesca)
G	CDP79023.1, CDP79024.1, CDP79026.1	Edestin 1 (*Cannabis sativ*a)
H	SNQ45158.1	Edestin 3 (*Cannabis sativa*)
I	KZN00901.1	Hypothetical protein DCAR_009655 (*Daucus carota* subsp. Sativus)
L	CDP79023.1, CDP79024.1, CDP79026.1	Edestin 1 (*Cannabis sativa*)
M	CDP79023.1, CDP79024.1, CDP79026.1	Edestin 1 (*Cannabis sativa)*
	EMS59070.1	Histone H4 (*Triticum urartu*)
N	ARF_MAIZE	ADP-ribosylation factor 1 (*Cannabis sativa)*
	CDP79023.1, CDP79024.1, CDP79026.1	Edestin 1 (*Cannabis sativa*)
	KZN00901.1	Hypothetical protein DCAR_009655 (*Daucus carota* subsp. Sativus)
	ABX09991.1	Actin 1, partial (*Ziziphus jujuba*)
	XP_022159030.1	Uncharacterized protein LOC111025474 (*Momordica charantia*)
	XP_023875501.1	GTP-binding protein rhoA (*Quercus suber*)
R	CDP79027.1	Edestin 2 (*Cannabis sativa*)

The fractionation of albumins allowed the partial depletion of edestin, the most abundant storage protein of hempseeds, improving the detection of other proteins involved in metabolic functions of seeds. The presence of Luminal binding protein 5 (BiP) and HSP70-like was detected in spot 32, while a protein disulfide isomerase-like and chaperonine 60 (RuBisCO large subunit-binding protein subunit alpha) were identified in spot 33.

These two spots are observed in the upper left side of the gel, and their intensity is clearly lower in seeds of Finola grown in the field of Viganella, as observed for other high molecular weight proteins. During seed maturation, storage proteins are folded within the endoplasmic reticulum (ER). This process is assisted by chaperones of the HSP70/BiP (luminal binding protein) family and protein disulfide isomerase (PDI). HSP70/BiP chaperones bind transiently to the nascent polypeptides and prevent protein misfolding under normal and stress conditions^10,21^, PDI catalyzes a thiol–disulfide exchange reaction leading to the formation of proper disulfide bonds that stabilize the structure of proteins^22^. Bips are implicated in the synthesis of storage proteins and stress response, however it was observed in transgenic rice that extreme BiP overexpression inhibited proper protein accumulation in protein bodies, resulting in floury and shrunken seeds, with low levels of seed storage proteins and starch contents compared with the wild type. On the contrary, PDI-overexpressing mutants present seeds with similar traits to the wild type^21^. PDI5 is necessary for seed development as it seems to be involved in the regulation of programmed cell death (PCD) by inhibition of Cys proteases during the trafficking to vacuoles. It was observed in *Arabidopsis thaliana* that the loss of PDI5 function led to premature PCD during embryogenesis and to the production of fewer, nonviable seeds^23^. Interestingly, two spots sharing a similar position to 32 and 33 spots in the 2D-gel were identified as HSP70 and luminal-binding protein 5 from albumin/globulin fractions of quinoa seeds as well, and resulted to increase under salinity stress^24^.

Besides the HSP70-like of spot 32, another form of HSP70 was identified in spot F, which was exclusive of Finola seeds grown in Viganella. This is probably a protein fragment because the observed molecular weight was lower than the theorethical one. Spots 13, 14, 15, 16 were identified as 18.5 kDa class I HSP protein like, and spot 47 was detected as 17.8 kDa class I HSP. These proteins are generally defined as “small Heat Shock Proteins” (sHSPs), characterized by a low molecular weight (12–40 kDa). Small HSPs present a short C-terminal sequence, a highly conserved α-cristallin domain (ACD), and a highly variable N-terminal region. These proteins act as chaperones which can form multimeric complexes and bind denatured proteins, preventing protein aggregation. Plant sHSPs belong to 6 classes, with class I including the cytoplasmic form^25,26^.

The members of Heat Shock Proteins (HSP) 70 (DnaK in prokaryotes) are conserved proteins involved in folding processes including the folding of nascent proteins, protein transport across membranes, and refolding of misfolded and aggregated proteins^27^. HSP70 and small heat shock proteins are implicated in heat sensitivity of plants and the overexpression of hsp70 often results in enhanced thermotolerance. Plastidial HSP70s are important for thermotolerance of germinating seeds, since plastids are involved in the assimilation of nitrogen and in the synthesis of lipids required during seed germination and growth^28,29^. Besides their role against heat stress, HSPs are also involved in seed development. Abscisic acid (ABA) is a plant hormone that plays a role in seed dormancy and germination control. It was observed that ABA auxotroph plants present enhanced germination and produce viviparous seeds^30^. A form of HSP70 was found to be necessary to establish the basal level of transcription of Abscisic acid (ABA)-responsive genes, suggesting a role in the long-term adaptation to drought and dehydration tolerance in vegetative tissues and/or seeds^31^. HSPs are highly expressed in mature and dry seeds, whereas they tend to disappear during germination^32^. 1-Cys peroxiredoxin (PER1) and glutathione S-transferase DHAR2-like were identified in spot 53, which is observable in all samples except for Finola seeds obtained from Viganella field. PER1 was also detected in spot 5, near to spot 53. The presence of spot 5 is more evident in seeds of Futura 75 obtained from the experimental fields of Crodo and Viganella. Spots 8 and 25 were identified as NADPH dependent aldehyde reductase 1. Spot 8 can be detected in the certified seeds of both varieties, while it decreases in Finola seeds from Viganella. On the contrary, seeds of Futura 75 grown in Viganella present both the spot 8 and a new spot (25), which was not observed in certified seeds. Reactive oxygen species (ROS) and reactive carbonyl species (RCS) are produced during seed storage, and might cause oxidative damage in seeds by oxidizing lipids to various aldehydes and ketones^33^. An effective protection against such reactions is especially important in seeds rich of linoleic and linolenic acid, which are sources of short chain carbonyls after peroxidation. NADPH- aldehyde reductases act in the detoxification of reactive carbonyls in plants catalyzing their reduction on α,β-unsaturated aldehydes and saturated aldehydes such as methylglyoxal^34^. Peroxiredoxins show antioxidant activity and can be divided into three classes: typical 2-Cys Prx, atypical 2-Cys Prx, and 1-Cys Prx (PER1), the latter being expressed in developing seeds. 1-Cys peroxiredoxin shows highest expression levels during the dehydration stages at the end of seed development and in the mature dry seed. It was suggested that PER1 is employed to sense and/or react to seed environmental conditions, preventing germination under unfavorable conditions^35^. Glutathione-S-transferases (GSTs) are a diverse family of proteins that detoxify xenobiotic and endobiotic compounds by conjugating GSH and protect against ROS-induced oxidative damage. One group is represented by glutathione dependent dehydroascorbate reductases (DHARs) which catalyzes the GSH-dependent reduction of dehydroascorbate (DHA) to ascorbate, a reaction implicated in plant redox homeostasis^36^. An increase of Glutathione-S-Tranferases (GST) and PER1 was observed in storage tolerant seeds respect with storage sensitive seeds, suggesting an enhanced seed tolerance to aging by an effective ROS detoxification using antioxidants such as GSTs and PER1^33^. Two spots sharing the same MW/pI parameters of spots 5 and 53 on 2D gel were identified as PER1 in soybean seeds and seedlings^37^, corresponding to a main protein form and a post-transationally modified form probably due to overoxidation, thus the two spots observed in hemp seeds could reflect a similar modification event.

The presence of PER1 in plants like soybean, rice and barley was reported to gradually decrease during germination. Observations on soybean demonstrated that the higher presence of PER1 in seedlings under flooding stress is not derived from newly synthesized protein, but is the result of delayed degradation^37^.

Spots 54 and 55 are located in the basic end of the lower molecular weight zone of the gel and present higher dimensions respect with other spots. This is congruent with the identification of the basic subunits of the three edestin types. In addition to these, 11 kDa late embryogenesis abundant protein (LEA) and CYP19-4 a cyclophilin with peptidyl-prolyl cis–trans isomerase (PPI) activity were identified in spot 54, while 60S ribosomal protein L12-3-like was detected in spot 55. Another LEA group 3-like of *C. sativa* was identified in spot 59, which is highly visible in certified seeds of Futura 75. Late embryogenesis abundant (LEA) proteins accumulate in seeds during the late developmental stage and represent an important response to adverse conditions such as dessiccation and freezing by acting as hydration buffers and stabilizing cell structures. LEA proteins can be classified into seven groups, of which 3 LEA proteins seem to be important for the establishment of tolerance to low temperature stress in seeds by protecting antioxidant enzymes such as peroxidase and superoxide dismutase^38^. The peptidyl-prolyl cis–trans isomerases (PPI) assist folding by catalyzing the cis–trans isomerization of Proline containing peptide bonds, a rate-limiting step in the folding of some proteins. It was observed in *Arabidopsis* plants that CYP19-4 is expressed during embriogenesis, assisting the folding and modulating the activity of EMB30/GNOM^39^.

Finally, spot 3 was identified as phosphoglycerate kinase by detecting two peptides from a cytosolic form from *Nicotiana tabacum* and *Arabidopsis thaliana* in the Swiss Prot database, which currently (23rd December 2020) contains only 19 reviewed proteins of *C. sativa*. Two more peptides were homology identified after searching the NCBInr database (Viridiplantae), each peptide belonging to a different organism. After Blast analysis, the same peptides were found in three hypothetical proteins of *C. sativa*: F8388_017609, G4B88_025149, G4B88_011096 (KAF4350031.1, KAF4347106.1, KAF4347679.1) which share a very similar sequence. The only difference observed is peptide R.VDLNVPLDDSLKITDDTR.I, which shows the R.VDLNVPLDD**N**L**T**ITDDTR.**V** sequence in *C. sativa*, with the different aminoacids in bold letters. The presence of T instead of K is consistent with the length of the observed peptide, otherwise it should be considered as a missed trypsin cleavage site. Phosphoglycerate kinase (PGK) is a monomeric enzyme of the glycolytic pathway which catalyzes the formation of ATP and 3-phosphoglycerate. On 2D maps, spot 3 is about 50 kDa and pI 5.5: phosphoglycerate kinase of *N. tabacum* and *A. thaliana* show similar parameters, whereas the three hypothetical proteins of *C. sativa* present longer sequences: according to ProtParam^40^ their MW/pI are around 92 kDa and 6.0 respectively. Due to the high similarity of the sequences, KAF4347679.1 was chosen among the three hypothetical proteins of *C. sativa* and was compared with the sequence of two phosphoglycerate kinases (chloroplastic and cytosolic) of *A. thaliana*. Interestingly, all the detected peptides are located in the second part of the sequence, while the GVTTIIGGGDSVAAVEK peptide is repeated twice in the sequence. In order to predict the cellular location of KAF4350031.1, this sequence was analyzed with DeepLoc 1.0, a tool for the prediction of eukaryotic protein subcellular localization^41^. Analysis of the entire sequence led to chloroplast prediction, while the analysis starting from aa 477 (MATK) led to cytosolic prediction. Since the sequence of Hypothetical protein F8388_017609 (KAF4350031.1) of *C. sativa* is the result of a genome assembly experiment, it is possible that this sequence includes two isoforms (chloroplastic and cytosolic) of phosphoglycerate kinase.

The spots at low molecular weight that were mainly observed in Finola samples from Viganella were frequenty identified as fragments of edestin, the main globulin of hemp.

### Total protein fraction

**Figure 5:**
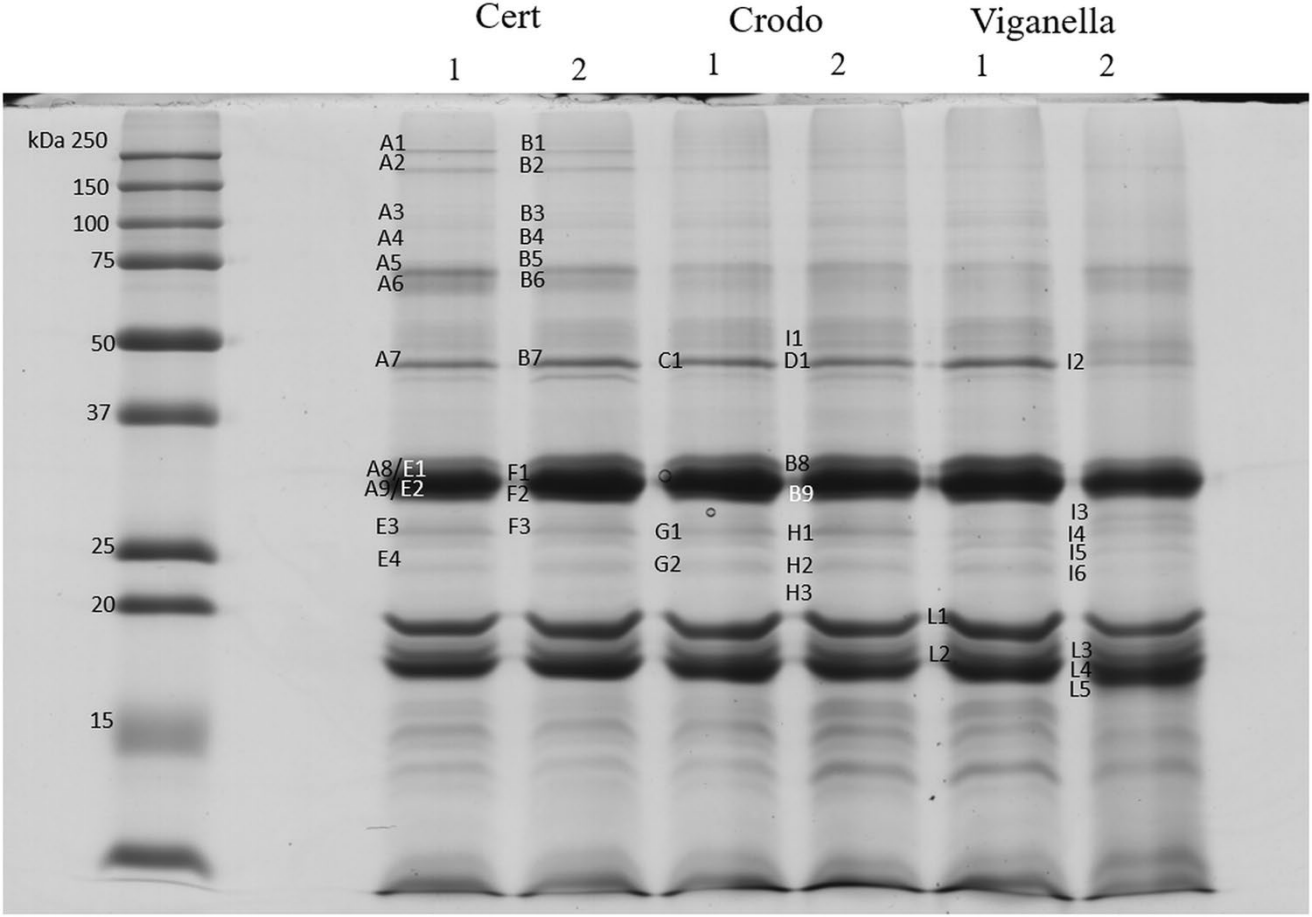
SDS-PAGE of total protein extracts of harvested seeds from the experimental fields of Crodo and Viganella and from certified (Cert) seeds of Futura 75 (1) and Finola (2) hemp varieties.

Globulins represent the primary storage proteins of legumes and oilseeds. With the aim to follow changes affecting the protein pattern of both globulins and metabolic proteins, a total protein extraction was performed and the proteins were separated by SDS-PAGE and 2D-PAGE. The profile of total proteins obtained after SDS-PAGE (Fig. 5) revealed the presence of different bands from 15 to > 75 kDa.

The three most intense bands are visible at 30, 20 and 18 kDa, corresponding to the molecular weight of the acidic and basic chains of edestin subunits, respectively. A less intense band is present around 45 kDa, with a marked decrease of intensity in seeds of Finola plants grown in the Viganella field. Other minor bands at a molecular weight higher than 75 kDa are more evident in certified seeds, while protein extracts from the experimental fields show more evident bands at low molecular weight (< 18 kDa) respect with certified seeds of both varieties.

The list of identified proteins from bands is reported in Table 4, whereas the peptide sequences obtained after MS/MS analysis are reported in supplementary table S2. As expected, the most intense bands present isoforms belonging to the three different types of edestin, with a variable number of identified peptides among different seed samples. The identified peptides from the 30 kDa band belong to the acidic subunit (A8, A9, E1, E2, F1, F2, B8, B9). The band at about 20 kDa (L1) contain peptides belonging to the basic subunit of edestin type1, while the two bands around 18 kDa (from L2 to L5) contain a mixture of peptides mainly belonging to the basic subunit of edestin type2 and type3. Few peptides of edestin 1, Vicilin C72-like and a heat shock 70 kDa protein-like were identified from bands at high molecular weight (> 75 kDa, A1-A6, B1-B6).

**Table 4:** Proteins identified after MS/MS analysis of total protein extracts from hemp seeds of Futura75 and Finola varieties analyzed by SDS-PAGE. Band identificative name (ID), accession number, protein name and organism are indicated.

ID	Accession number	Protein name
A1	CDP79023.1	Edestin 1 (*Cannabis sativa*)
	XP_030508280.1	Vicilin C72-like (*Cannabis sativa*)
A2	CDP79023.1	Edestin 1 (*Cannabis sativa*)
A3	CDP79023.1	Edestin 1 (*Cannabis sativa*)
A4	CDP79023.1	Edestin 1 (*Cannabis sativ*a)
A5	CDP79023.1	Edestin 1 (*Cannabis sativa*)
A6	CDP79023.1	Edestin 1 (*Cannabis sativa*)
	XP_030478962.1	Heat shock 70 kDa protein-like (*Cannabis sativa*)
	CDP79027.1	Edestin 2 (*Cannabis sativa*)
A7	XP_030508280.1	Vicilin C72-like (*Cannabis sativa*)
	XP_030493178.1	Sucrose-binding protein-like (*Cannabis sativa*)
	CDP79023.1	Edestin 1 (*Cannabis sativa*)
	AAB01374.1	Beta-conglycinin storage protein (*Glycine max*)
A8	CDP79028.1	Edestin 2 (*Cannabis sativa*)
	CDP79023.1	Edestin 1 (*Cannabis sativa*)
	SNQ45160.1	Edestin 3 (*Cannabis sativa*)
	XP_004232705.1	Lactoylglutathione lyase GLX1 (*Solanum lycopersicum*)
	XP_030508280.1	Vicilin C72-like (*Cannabis sativa*)
	XP_030493178.1	Sucrose-binding protein-like (*Cannabis sativa*)
A9	SNQ45160.1	Edestin 3 (*Cannabis sativa*)
	CDP79023.1	Edestin 1 (*Cannabis sativa*)
	CDP79028.1	Edestin 2 (*Cannabis sativa*)
	XP_030506286.1	NADPH-dependent aldehyde reductase 1, chloroplastic-like (*Cannabis sativa*)
	XP_030477762.1	Aspartic proteinase A1-like (*Cannabis sativa*)
	EEF43857.1	Lactoylglutathione lyase, putative (*Ricinus communis*)
	XP_030495935.1	LOW QUALITY PROTEIN: 3-oxoacyl-(acyl-carrier-protein) reductase 4-like (*Cannabis sativa*)
	XP_030508280.1	Vicilin C72-like (*Cannabis sativa*)
	XP_030493178.1	Sucrose-binding protein-like (*Cannabis sativa*)
B1	XP_030508280.1	Vicilin C72-like (*Cannabis sativa*)
	CDP79023.1	Edestin 1 (*Cannabis sativa*)
B3	CDP79023.1	Edestin 1 (*Cannabis sativa*)
B5	CDP79023.1	Edestin 1 (*Cannabis sativa*)
B6	CDP79023.1	Edestin 1 (*Cannabis sativa*)
B7	XP_030508280.1	Vicilin C72-like (*Cannabis sativa*)
	CDP79023.1	Edestin 1 (*Cannabis sativa*)
	XP_030493178.1	Sucrose-binding protein-like (*Cannabis sativa*)
	CDP79028.1	Edestin 2 (*Cannabis sativa*)
B8	CDP79028.1	Edestin 2 (*Cannabis sativa*)
	CDP79023.1	Edestin 1 (*Cannabis sativa*)
	SNQ45160.1	Edestin 3 (*Cannabis sativa*)
	XP_030493178.1	Sucrose-binding protein-like (*Cannabis sativa*)
	XP_030508280.1	Vicilin C72-like (*Cannabis sativa*)
B9	SNQ45160.1	Edestin 3 (*Cannabis sativa*)
	CDP79023.1	Edestin 1 (*Cannabis sativa*)
	CDP79027.1	Edestin 2 (*Cannabis sativa*)
	XP_030506286.1	NADPH-dependent aldehyde reductase 1, chloroplastic-like (*Cannabis sativa*)
	XP_006348126.1	Lactoylglutathione lyase GLX1 (*Solanum lycopersicum*)
	XP_030508280.1	Vicilin C72-like (*Cannabis sativa*)
	XP_030481474.1	Mitochondrial outer membrane protein porin of 34 kDa-like (*Cannabis sativa*)
C1	XP_030508280.1	Vicilin C72-like (*Cannabis sativa*)
	PNY00005.1	Tubulin alpha-3 chain-like protein (*Trifolium pratense*)
	GAX73914.1	Hypothetical protein CEUSTIGMA_g1364.t1 (*Chlamydomonas eustigma*)
	CDP79023.1	Edestin 1 (*Cannabis sativa*)
	XP_030508778.1	ADP-ribosylation factor 1(*Cannabis sativa*)
	XP_030493178.1	Sucrose-binding protein-like (*Cannabis sativa*)
D1	XP_030508280.1	Vicilin C72-like (*Cannabis sativa*)
	CDP79023.1	Edestin 1 (*Cannabis sativa*)
	XP_030493178.1	Sucrose-binding protein-like (*Cannabis sativa*)
	CDP79028.1	Edestin 2 (*Cannabis sativa*)
E1	AAO45103.1	Beta-conglycinin alpha' subunit, partial (*Glycine max)*
	CDP79023.1	Edestin 1 (*Cannabis sativa*)
	CDP79027.1	Edestin 2 (*Cannabis sativa*)
	KHN10743.1	Glycinin G2 (*Glycine soja*)
E2	SNQ45160.1	Edestin 3 (*Cannabis sativa*)
	CDP79028.1	Edestin 2 (*Cannabis sativa*)
	XP_030506286.1	NADPH-dependent aldehyde reductase 1, chloroplastic-like (*Cannabis sativa*)
E3	CDP79023.1	Edestin 1 (*Cannabis sativa*)
	SNQ45153.2	7S vicilin-like protein (*Cannabis sativa*)
	CDP79028.1	Edestin 2 (*Cannabis sativa*)
F1	CDP79023.1	Edestin 1 (*Cannabis sativa*)
	CDP79027.1	Edestin 2 (*Cannabis sativa*)
F2	XP_030508280.1	Vicilin C72-like (*Cannabis sativa*)
	CDP79023.1	Edestin 1 (*Cannabis sativa*)
	SNQ45160.1	Edestin 3 (*Cannabis sativa*)
	CDP79028.1	Edestin 2 (*Cannabis sativa*)
	XP_030493178.1	Sucrose-binding protein-like (*Cannabis sativa*)
	XP_030506286.1	NADPH-dependent aldehyde reductase 1, chloroplastic-like. (*Cannabis sativa*)
G1	SNQ45160.1	Edestin 3 (*C. sativa*)
	CDP79023.1	Edestin 1 (*Cannabis sativa*)
	CDP79028.1	Edestin 2 (*Cannabis sativa*)
	SNQ45153.2	7S vicilin-like protein (*Cannabis sativa*)
	XP_030506286.1	NADPH-dependent aldehyde reductase 1, chloroplastic-like (*Cannabis sativa*)
H1	CDP79023.1	Edestin 1 (*Cannabis sativa*)
	SNQ45158.1	Edestin 3 (*Cannabis sativa*)
H2	CDP79023.1	Edestin 1 (*Cannabis sativa*)
	SNQ45153.2	7S vicilin-like protein (*Cannabis sativa*)
	CDP79027.1	Edestin 2 (*Cannabis sativa*)
I1	SNQ45158.1	Edestin 3 (*Cannabis sativa*)
	CDP79023.1	Edestin 1 (*Cannabis sativa*)
I2	CDP79023.1	Edestin 1 (*Cannabis sativa*)
	XP_030508280.1	Vicilin C72-like (*Cannabis sativa*)
	XP_030493178.1	Sucrose-binding protein-like (*Cannabis sativa*)
I3	XP_030508280.1	Vicilin C72-like (*Cannabis sativa*)
	CDP79023.1	Edestin 1 (*Cannabis sativa*)
	XP_030493178.1	Sucrose-binding protein-like (*Cannabis sativa*)
	SNQ45160.1	Edestin 3 (*Cannabis sativa*)
I4	CDP79028.1	Edestin 2 (*Cannabis sativa*)
	CDP79023.1	Edestin 1 (*Cannabis sativa*)
	SNQ45160.1	Edestin 3 (*Cannabis sativa*)
	SNQ45153.2	7S vicilin-like protein (*Cannabis sativa*)
	BAK03519.1	Predicted protein (*Hordeum vulgare* subs. Vulgare)
I5	CDP79023.1	Edestin 1 (*Cannabis sativa*)
	CDP79028.1	Edestin 2 (*Cannabis sativa*)
	XP_004232705.1	Lactoylglutathione lyase GLX1 (*Solanum lycopersicum*)
	XP_030506286.1	NADPH-dependent aldehyde reductase 1, chloroplastic-like (*Cannabis sativa*)
	XP_030508280.1	Vicilin C72-like (*Cannabis sativa*)
	XP_030493178.1	Sucrose-binding protein-like (*Cannabis sativa*)
I6	XP_030508280.1	Vicilin C72-like (*Cannabis sativa*)
	XP_030493178.1	Sucrose-binding protein-like (*Cannabis sativa*)
	CDP79023.1	Edestin 1 (*Cannabis sativa*)
	SNQ45153.2	7S vicilin-like protein (*Cannabis sativa*)
	CDP79028.1	Edestin 2 (*Cannabis sativa*)
L1	CDP79023.1	Edestin 1 (*Cannabis sativa*)
L2	CDP79023.1	Edestin 1 (*Cannabis sativa*)
	SNQ45158.1	Edestin 3 (*Cannabis sativa*)
L3	SNQ45158.1	Edestin 3 (*Cannabis sativa*)
	CDP79023.1	Edestin 1 (*Cannabis sativa*)
L4	CDP79028.1	Edestin 2 (*Cannabis sativa*)
	SNQ45158.1	Edestin 3 (*Cannabis sativa*)
	CDP79023.1	Edestin 1 (*Cannabis sativa*)
L5	CDP79023.1	Edestin 1 (*Cannabis sativa*)
	CDP79028.1	Edestin 2 (*Cannabis sativa*)
	SNQ45158.1	Edestin 3 (*Cannabis sativa*)

Based on the sedimentation coefficients, globulins are classified as 7S (vicilin-like) and 10-12S (legumin-like) globulins. Legumin-like globulins are hexamers (300–370 kDa) composed of six subunits of about 50–60 kDa, where each subunit is post-translationally cleaved to obtain acidic (N-terminal, 30 kDa) and basic (C-terminal, 20–22 kDa) chains, linked by a disulfide bond formed in the precursor protein. Vicilin-like proteins, the second group of seed globulins, are trimers of 150–190 kDa composed of subunits of different molecular weight (50–70 kDa). Unlike the majority of 10-12S globulins, 7S globulins are generally glycosylated and can not form disulfide bonds^10^. Edestin is the 11S globulin contained in protein bodies of hempseeds, showing a structure similar to that of soy glycinin^42^. Previous works on hemp protein isolates analyzed by SDS-PAGE detected the acidic subunit (AS) of edestin as a homogeneous band of 34 kDa, while the basic subunit (BS) in two different bands of about 20 and 18 kDa. Therefore the molecular weight of edestin in its hexameric form is estimated to be 300 kDa^43–45^. Recently, genes encoding for different isoforms of edestin were identified from Carmagnola and Futura hemp cultivars: CsEde1, CsEde2 and CsEde3, where CsEde1and CsEde3 genes are co-localized on the same DNA segment^46,47^. As inferred from the deduced protein sequences, the two isoforms of edestin type3 are particularly rich of sulphurated aminoacids, showing higher Cys content respect with edestin type1 and type2, whereas Met content is similar to that of edestin type2^47^. Other minor storage proteins of hemp seeds are represented by 7S vicilin and 2S albumin. Two genes encoding for 2S albumin clustered together in a tail-to-head array, and one gene encoding for a 7S vicilin-like were identified and characterized. The deduced 7S protein is a single polypeptide of 53.51 kDa containing two cupin_1 domains, four putative glycosylation sites within the cupin domains, and no inter-chain disulfide bonds^47^. The presence of a 7S minor polypeptide of about 48.0 kDa was observed on polyacrylamide gels^43–45^. We identified the following proteins belonging to the 7S family: vicilin C72-like, sucrose-binding protein-like and 7S vicilin-like.

Vicilin C72-like was detected mainly at 45 kDa from a defined band which was well defined in all samples except for Finola extracts obtained from the Viganella field, showing a marked decrease of intensity. Vicilin C72 was detected with the highest number of peptides in bands from certified seeds of both varieties (A7, B7) and with a progressively lower number of peptides in samples from Crodo (C1, D1) and the lowest number of peptides detected in Finola samples from Viganella (I2). The identified peptides belong to the protein sequence spanning from aa 467 to 824. Vicilin C72-like was also detected with a high number of peptides in 30 kDa bands, mainly F2 and I3, covering the protein sequence from aa 514–824. This protein contains two cupin_7S_vicilin-like domains spanning from aa 438 to 622 and from aa 643 to 812, and a Vicilin_N superfamily located at the N-terminal of cupin domain, from aa 308 to 383 (accession cd02244, cd02245 and cl23732 in the conserved domain database of NCBI (http://www.ncbi.nlm.nih.gov/Structure/cdd). Peptides from the Vicilin_N_superfamily domain were not detected from this band and the MW calculated with the ProtParam tool^40^ is consistent with the apparent molecular weight observed on the gel, suggesting post/co-translational modification events. Sucrose-binding protein-like (SBP) contains two cupin_7S_vicilin-like domains spanning from aa 102 to 275 and from 305 to 467. SBP was detected at 45 kDa mainly in certified samples (A7, B7), while fewer matches were obtained from samples of the experimental fields (C1, D1, I2). The identified peptides cover the protein sequence from aa 170 to 474. SBP was also detected at 32–35 kDa (A8, A9, B8, F2) and, in samples of Finola Viganella, at lower molecular weight (25–30 kDa, I3, I5, I6). The peptides that were detected in these bands cover protein sequence from aa 204 to 474, with an estimated MW of 31 kDa.

Finally, the 7S vicilin-like protein was identified in bands around 25–30 kDa (E3, G1, H2, I4, I6) of samples from both experimental fields and certified seeds. The analysis of conserved domains revealed the presence of two cupin domains from aa 66 to 226 and from aa 321 to 470.

Vicilin C72-like, sucrose-binding protein-like and 7S vicilin-like all contain two cupin-like domains with a beta-barrel folding typical of 7S seed storage proteins such as beta-conglycinin, phaseolin, and a sucrose binding protein of soybean. Beside storage function, these proteins can act as antioxidants and seem to play defensive role in germinating seeds. These proteins are cleaved into fragments before and during the germination process, with some of them presenting antimicrobial activity, as observed in macadamia seeds^48^.

Finally, despite the low reducing power of the SDS-PAGE technique, metabolic proteins less abundant than seed storage proteins, were identified. NADPH dependent aldehyde reductase 1, already detected in spot 8 and 25 of the albumin fraction, was found at 32–35 kDa (A9, B9, E2, F2) from certified and Crodo samples of both varieties, and at 25 kDa (I5) from Finola samples of Viganella. Lactoylglutathione lyase was detected at 30 kDa (A9, B9) and at 25 kDa (I5). This enzyme is involved in the first step of methylglyoxal detoxification forming S-lactoylglutathione. The ADP-rybosylation factor 1 (ARF1) was detected in the band C1 (total extracts), spot 3 and spot N (albumins) of Finola samples from Viganella field. ARF1 is a GTP-binding protein involved in the formation of vesicles, playing a role in coat protein complex I (COPI)-mediated retrograde trafficking. ARF1 is targeted to the Golgi and endosomes, with the active form associated to the membrane, whereas the inactive form of ARF1 is cytosolic^49^.

### Spot analysis of total protein extracts

**Figure 6:**
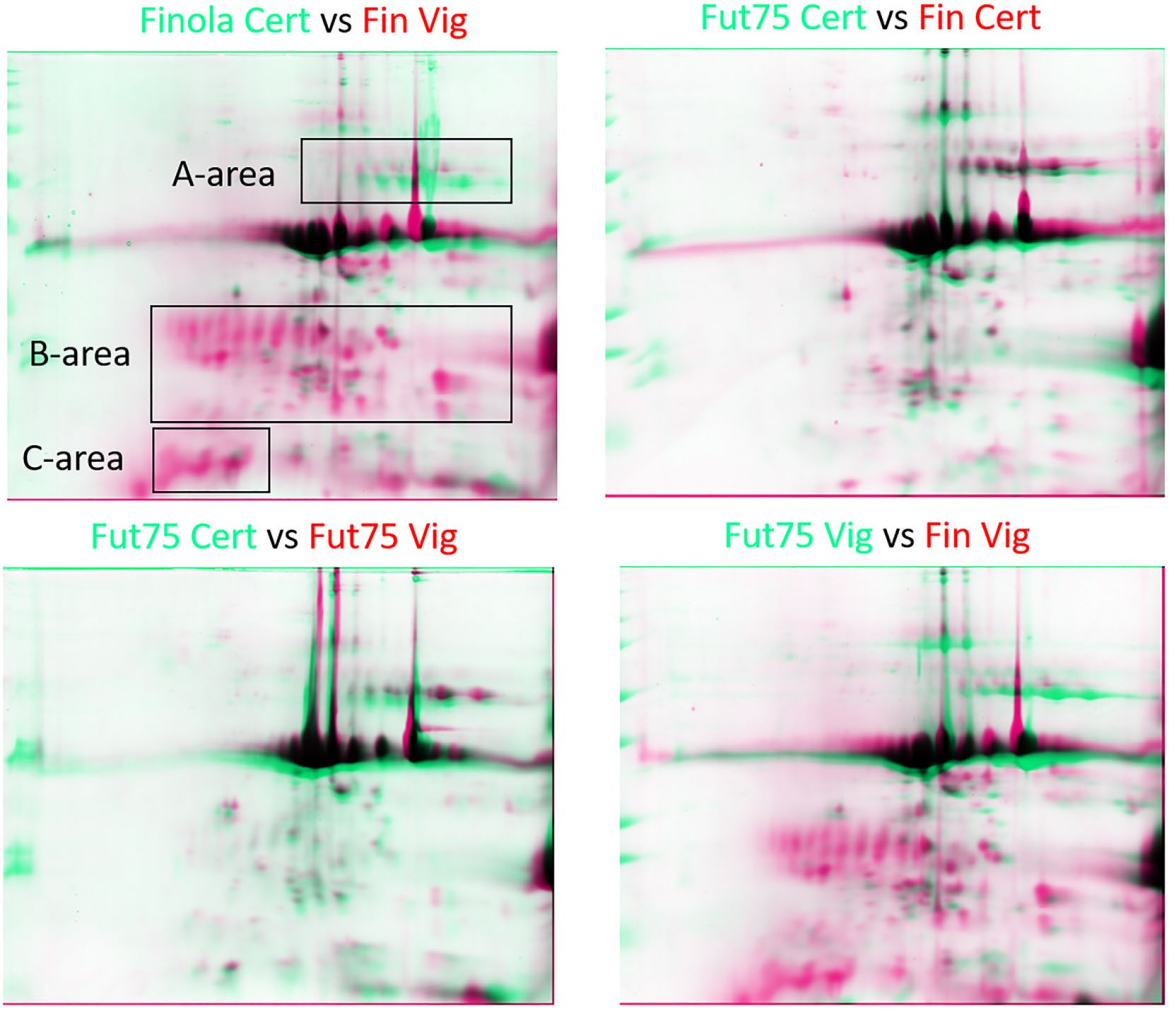
Overlapping images from 2D-gels of total protein extracts: certified and harvested seeds from the experimental field of Viganella, Finola and Futura 75 varieties. The three areas (**A**, **B**, **C**) define gel zones mainly affected by changes in protein distribution.

2D-gels from total protein extracts obtained from seeds of Futura 75 and Finola grown in Viganella were compared with those obtained from certified seeds, to get information about single protein changes. The spots resulting varying after image analysis are shown in figure S7 and supplementary table S3. Overlapping maps of different samples are depicted in Fig. 6, where three gel areas (A, B, C) highlight the parts which were mainly interested by changes in protein distribution. Protein identifications obtained after MS analysis of varying spots are listed in Table 5.

**Table 5:** MS/MS identifications from total protein extracts after 2D-PAGE analysis. Different gel areas, database accession code (AC), protein name and organism, theoretical molecular mass and peptide sequences are indicated.

Zone	AC	Protein name	Mass	Peptide sequence
A	KAF4346956.1 (XP_030493178.1)	Hypothetical protein G4B88_020994 (Sucrose-binding protein-like) (*C. sativa*)	55,771	GAMSAPFYNSR SQNEEYFFPGPR
	KAF4383765.1 (XP_030508280.1)	Hypothetical protein G4B88_020087 (vicilin C72) (*C. sativa*)	110,182	GTINVVR TVGFGVNAR FYEVTPEQNK ADVIVVPAGSTVYMTNQDNK TTVLVMVVEGTGR LTGEQRQGQRQGQR
B	SNQ45157.1	Edestin 1 (*C. sativa*)	58,810	GQGQGQSQGSQPDR
	SNQ45155.1	Edestin 2 (*C. sativa*)	56,350	LQVVDDNGR
C	SNQ45155.1	Edestin 2 (*C. sativa*)	56,350	GEDLQIIAPSR SEGASSDEQHQK RESGEQTPNGNIFSGFDTR + Deamidated (NQ)

A significant change in protein abundance was reported for 19 spots. The most significant changes were observed in Finola seeds from Viganella, where it was observed the decrease of spots at high molecular weight (73, 74, 75, 76) identified as Vicilin C72 and sucrose binding protein, and an increase of spots 9, 12, 22, 23, 33, 38, 39, 41, 42, 49. These spots are included in the low molecular weight part (denoted as “B” area) of figure 6, which also contains spots in the 18–25 kDa area that were uniquely observed in Finola seeds of Viganella. Among those with increasing intensity, spots 22, 23, 38, 39 and 49 were identified as edestin. The spots 22, 23, 38 and 39 could be ascribed to fragments of edestin, since the observed MW/pI are distant from the theoretical ones, which are estimated around 21 kDa/9.5 for the basic subunit, and 35 kDa with different pI for edestin 1 (6.36), edestin 2 (7.76) and edestin 3 (5.67) acidic subunits.

A decrease of 11S globulins was also observed in seeds of *Chenopodium quinoa* plants affected by salt stress as a result of protein degradation, and a concomitant increase of branched-chain amino acids (V, L, I) with a possible role as osmolytes^24^. The increase of fragmentation observed for storage proteins such as edestin is an interesting feature, since there is a growing body of evidence concerning the presence of bioactive peptides in hempseeds. Several studies have reported antioxidant, antihypertensive, antiproliferative, hypocholesterolemic, anti-inflammatory and neuroprotective effects from hempseed hydrolysates^4^. The bioactivity extent seems to be correlated to peptide length and sequence. It was observed that hydrophobic peptides are associated with antioxidant effects and the presence of a branched-chain, hydrophobic aa located at the N-terminal side is associated with a strong ACE-inhibitory activity. A previous study^50^ identified bioactive peptides obtained from simulated gastrointestinal digestion of hempseed proteins. At least part of them are derived from edestin: FEQL, WSY and IPAGV are located in the acidic subunit of edestin 1, LQL is in the basic subunit of edestin types 1 and 2, while YNL peptides is present in the basic subunit of the three types of edestin. Of these, IPAGV peptide was found to have a strong effect in lowering systolic blood pressure, probably due to resistance to inactivation after digestion and strong binding to target enzymes.

The studies conducted so far on hemp bioactive peptides have been performed starting from hemp seed protein isolates^51^ in fact only one study was performed directly on the whole seed^52^. Our results highlight the variations of protein profile under different environmental conditions and we have detected the presence of naturally produced peptides from storage proteins. The environment was able to directly modify the protein pattern of the seed to a different degree according to different cultivars, possibly improving nutritional properties of seeds. This feature could be exploited for the valorization of the consumption of dehulled hempseed.

## Conclusions

In this work it was observed that mountain environment affected some traits of Finola seeds such as fatty acid and protein profiles. These changes were not predictable by the sole comparison of certified seeds from Futura 75 and Finola cultivars. The fatty acid profile confirmed a high PUFA content in both cultivars from mountain environments, and the ω6/ω3 ratio of Futura 75 was in the range established to be ideal for human consumption. Interestingly, harvested Finola seeds from both experimental fields contained lower amount of ω3, with a consequently higher ω6/ω3 ratio. Moreover, these seeds presented a significant increase of the monounsaturated acid Cis-11-eicosenoic acid (C20:1), which is usually scarce in higher plants and is frequently used in industry as a moisturizing agent. A similar increase was observed in fungi grown at low temperature, and a correlation of EA with elevation was found in *Sapindus* spp. seeds, supporting a possible plasticity trait for environmental adaptation in Finola plants. Protein analysis revealed a decrease in the protein content of Finola seeds from the field of Viganella. The choice of different methods of protein extraction allowed the detection of less abundant proteins with metabolic functions, mainly involved in protein folding and response to oxidative stress. The most abundant protein that was detected in hempseeds was the globulin edestin, but other *C. sativa* specific globulins belonging to the 7S family were identified: Vicilin C72-like, sucrose-binding protein-like and 7S vicilin-like. These proteins could also be involved as antioxidants and seem to play defensive role in germinating seeds, when they are cleaved into fragments.

The changes observed in the protein pattern suggest an early germination onset of Finola seeds from Viganella, which are characterized by an extensive fragmentation of storage proteins and a decrease of intensity of proteins related to the inhibition of germination or in the mainteinance of seed dormancy. The increase of seed storage fragments observed in these seeds may represent an interesting feature that can be exploited as an extra source of antioxidant and bioactive peptides, confirming hempseeds as a valued food.

## Methods

### Experimental fields and sample collection

The experimental areas are located in the Verbano Cusio Ossola, with a mean elevation of 570 m above sea level (a.s.l.). This area falls within Alpi Pennine and Lepontine, in the North-Western Alps subsection (Western Alps Section) and belongs to the Temperate semicontinental bioclimate^53^.

This study describes the features of hempseeds from plants grown in two experimental fields, named “Viganella” and “Crodo” from their locations. The experimental field “Viganella” is a plot of  25 m^2^ obtained from terraced mountainside in the municipality of Viganella, a small village (hamlet of Borgomezzavalle) surrounded by the mountains in the middle of Valle Antrona (Latitude 46°03′09.3" N, Longitude 8°11′37.0" E, elevation 583 m a.s.l.). The experimental field “Crodo” is a flat plot of 120 m^2^ with South-East exposure (Latitude 46°13′36" N, Longitude 8°19′25" E, elevation 560 m a.s.l.) in the city of Crodo located in Valle Antigorio. The fields were not fertilized or irrigated during the growing season. The two varieties were sown with a pattern of 20 cm between rows and at intervals of 15 cm within each row.

Measurements of the temperature (°C) and rainfall (mm) were provided by the meteorological sensors from a regional network with locations at both sites (ARPA Piemonte, Agenzia Regionale per la Protezione Ambientale). The meteorological sensor nearest to Viganella is “Alpe Cheggio”, in the municipality of Antrona Schieranco (Latitude 46°05′06″ N, Longitude 08°06′56″ E, 1460 m a.s.l.), while the experimental field “Crodo” presents a local meteorological station. Certified seeds of the two varieties Futura 75 (FUTURA 75 FR 484520 AA COD. B 174613 02/2018) and Finola (FINOLA DE 166-2700754 11-2016) were used for the sowing, which took place in the last two weeks of May 2018. The seeds were collected at maturity by manually harvesting then left to dry in a cool and dry room for a couple of weeks.

### Seed weight

The seed weight was assessed for each variety and source (certified or harvested seeds from the experimental fields) by weighing a sample of 50 seeds, randomly selected, using an analytical balance (Quintix, Sartorius) and repeated in triplicate.

### Extraction and quantification of photosynthetic pigments

The photosynthetic pigments were extracted from 50 hempseeds and two biological replicates were considered. The seeds were ground in a mortar, then 0.1 g were taken and placed in a screw cap tube with 4 ml of 90% acetone. The tubes were wrapped with aluminum foil and stored at 4 °C for 16 h. After incubation, the tubes were vortexed at room temperature and the precipitate was left to settle, then 1 ml of the supernatant was transferred to a quartz cuvette (1 cm optical path) closed with a Teflon cap to prevent acetone evaporation. The samples were read at the spectrophotometer (SAFAS UV mc2 180–1050 nm), using the SAFAS SP2000 software (6.7 version) for data acquisition and processing. A wavelength scan from 350 to 700 nm was performed for each sample. Subsequently, the chlorophyll was transformed into pheophytin by acidifying the solution with 10 μl of HCl 0.1 N. Ten minutes after, a second spectrum was measured. The chlorophyll content was calculated using the equation described by Steinman et al.^54^ while total carotenoids were estimated using the Züllig Equation^55^.

### Seed fatty acids composition

Seed samples of the investigated varieties were ground using superfine grinding extractor—intensive vibrational mill (Model MM400, Retsch GmbH, Haan, Germany). To obtain a representative seed powder, a 50 ml jar with 20 mm stainless steel balls at a frequency of 25 Hz for 1 min was used. Lipid extraction^56^ was performed using 7.0 g of powdered seeds. The seed oil was extracted by a Soxhlet extractor and petroleum ether for 6 h at 60 °C. n-Hexane was used as the solvent and following the extraction method oil was separated from n-hexane using a rotator apparatus. The fatty acid composition of hemp seeds was determined using GC. In this method, the fatty acids were turned volatile using the method of methyl esterification^57^. The prepared solution was injected into a GC Trace Ultra (ThermoFisher Scientific) equipped with a flame ionization detector (FID), with the following specifications. Capillary column RTX-2560 (100 m × 0.25 mm id, 0.20 μm); the carrier gas was nitrogen, with the purity of 99.9%. The injector and the detector temperature were 260 and 280 °C, respectively. The oven temperature was kept at 100 °C for 5 min and increased to 240 °C at the rate of 4 °C per minute and maintained at 240 °C for 30 min^58,59^, The chromatographic profiles of analyte were elaborated with an Azur Software (Analytical Technology, Brugherio, Italia). Identification and quantitative evaluation of fatty acids was realized comparing retention times and areas with the ones of FAMEs (Fatty Acid Methyl Esters) standard mixes. All the analyses were done in three biological replicates.

### Protein extraction

Protein fractions were obtained from seeds produced by Finola and Futura 75 cultivars grown in the two experimental fields (Crodo and Viganella) following the sequential and the total protein extraction methods^60,61^. Protein extracts obtained from certified seeds were used as a reference control. The sequential method was applied to recover proteins showing different solvent solubility. The albumin fraction was obtained by grinding 1.0 g of hempseeds in a mortar at the temperature of 4 °C and mixed with a solution of 10 mL of ultrapure water containing 1% of protease inhibitor cocktail for plant cell and tissue extracts (P9599, Sigma Aldrich). The mixture obtained was transferred to Teflon tubes and left at a temperature of 4 °C for 2 h, vortexing occasionally (ZX3, Advanced Vortex Mixer, VELP Scientifica). The sample was centrifuged at 10,000 × g/4 °C for 15 min. Then the supernatant has been transferred into new tubes and stored at 4 °C. A second extraction of the pellet was done by adding 10 mL of ultrapure water with 1% of protease inhibitor cocktail and repeating the procedure described. The second supernatant was added to the previous one and stored at − 20 °C until use. The pellet obtained from the previous step was used to recover the globulin fraction, performing its solubilization in 10 mL of Tris–HCl 50 mM pH 8.0 and NaCl 0.3 M and incubating for two hours at 4 °C. Then the homogenate was centrifuged at 10,000 × g, 4 °C for 15 min. The supernatant was transferred to a new tube and stored at − 20 °C, while the pellet was used to recover the prolamin-like fraction. The pellet was solubilized with 10 mL of 70% ethanol and 0.2% 2-mercaptoethanol, left for 3 h at 4 °C and centrifuged at 10,000 × g, 4 °C for 30 min. The supernatant was transferred into a new tube and stored at − 20 °C. The pellet was dried (Concentrator plus, Eppendorf) and used to extract the final fraction of glutelin-like proteins. The pellet was mixed with 10 mL of NaOH 0.1 M, left at 4 °C for 2 h and then centrifuged at 10,000 × g for 30 min at 4 °C. The supernatant was stored at − 20 °C until use. Protein fractions obtained from the sequential extraction method were then precipitated in 10% Trichloroacetic acid (TCA)/Acetone and solubilized in 7 M urea, 2 M thiourea, 4% w/v CHAPS, 100 mM dithiothreitol (DTT), IPG-buffer (pH 3–10), in order to remove interfering salts for 2D-PAGE analyses.

Total proteins were extracted as indicated in^60^. Hempseeds (1 g) were ground in a mortar at 4 °C and mixed with 20 ml of 10% TCA in cold acetone (− 20 °C), 20 mM DTT and 1% protease inhibitors cocktail (Sigma). The homogenate was filtered, transferred into tubes and incubated overnight at − 20 °C to allow complete protein precipitation. The samples were centrifuged (18,000 × g, 1 h, 4 °C), and the pellet was washed three times with cold acetone and finally dried. The protein pellet was solubilized 1 h at room temperature in 7 M urea, 2 M thiourea, 4% w/v CHAPS, 100 mM DTT, IPG-buffer (pH 3–10), occasionally vortexing.

The protein content of different extracts was estimated using the Bradford assay^62^ with bovine serum albumin (BSA) as the protein standard.

### Protein analysis

Hempseed proteins resulting from sequential or total protein extractions were analyzed by sodium dodecyl sulphate–polyacrylamide gel electrophoresis (SDS-PAGE) and two-dimensional polyacrylamide gel electrophoresis (2D-PAGE) followed by mass spectrometry (LC–MS/MS) protein identification.

For SDS-PAGE, 10 µg of protein extracts were mixed with Laemmli buffer (2% w/v SDS, 10% glycerol, 5% 2-mercaptoethanol, 62 mM Tris–HCl pH 6.8) and loaded onto 10 × 8 cm vertical 12% polyacrylamide gels.

For 2D-PAGE, 150 µg of albumin, globulin, glutelin-like and total protein extracts were mixed with rehydration buffer (9 M Urea, 4% CHAPS) and submitted to isoelectric focusing (IEF) using linear IPG-strips (pH 3–10, 7 cm, BioRad) using the following parameters: 7 h of passive rehydration followed by 7 h at 50 V, 15 min at 250 V with rapid voltage increase, 1 h with a gradual voltage increase up to 4000 V and focusing at 4000 V for a final amount of 20,000 V/hours. The strips were equilibrated in presence of SDS performing a reducing step with DTT (15 min) followed by an alkylation step with iodoacetamide (10 min) and finally loaded to a 12% polyacrylamide gel. A plug of protein standards (Precision Plus Protein Dual Color, Biorad) was loaded in order to estimate the apparent molecular weight of proteins, and the strips were sealed with 1% agarose solution. Both SDS-PAGE and 2D-PAGE second dimension were performed at 15 mA for 30 min and 30 mA with a Mini Protean System (BioRad). The running buffer was 25 mM Tris–HCl, 200 mM glycine, 0.1% w/v SDS. Gel staining was performed with Colloidal Coomassie brilliant blue G250 and the gel image was acquired by a GS-900 densitometer using the software ImageLab (BioRad).

### Spot analysis of total protein extracts

The 2DE gels of total protein extracts obtained from certified and Viganella seeds of both varieties were analyzed using PDQuest software (BioRad) in order to detect spot differences among the tested samples. Four gels from three different biological replicates were compared for the analysis. Statistical analysis was performed using one-way ANOVA and differences were considered significant with p-value < 0.05.

### Protein digestion and MS analysis

Bands and spots of interest observed from albumin and total protein extracts were manually excised from the gels and trypsin digested as described in^63^. The peptide digests were desalted on the Discovery DSC-18 solid phase extraction (SPE) 96-well Plate (25 mg/well) (Sigma-Aldrich Inc., St. Louis, MO, USA) prior the mass spectrometry analysis. The LC–MS/MS analyses were performed by a micro-LC Eksigent Technologies (Dublin, USA) system and the mass spectrometer worked in data dependent acquisition mode (DDA)^64^. The mass spectrometry files were searched using Mascot v. 2.4 (Matrix Science Inc., Boston, MA, USA) with the following search parameters: trypsin as the digestion enzyme, 2 missed cleavages allowed, C carbamidomethylation (fixed modification), M oxidation and N/Q deamidation (variable modifications). A search tolerance of 50 ppm was specified for the peptide mass tolerance and 0.1 Da for the fragment mass.

## Supplementary information

Supplementary information.
