# Supplementary material for: Biochemical aspects of seeds from Cannabis sativa L. plants grown in a mountain environment
Source: Sci Rep. 2021 Feb 16;11:3927. doi: 10.1038/s41598-021-83290-1 (PMC7887209; doi:10.1038/s41598-021-83290-1)
Supplement: Supplementary file 1 — Supplementary information. [file 41598_2021_83290_MOESM1_ESM.pdf]

Biochemical aspects of seeds from *Cannabis sativa* L. plants grown in a mountain environment.

Chiara Cattaneo[1,*], Annalisa Givonetti[1], Valeria Leoni[2,3], Nicoletta Guerrieri[4], Marcello Manfredi[5], Annamaria Giorgi[2,3], Maria Cavaletto[1]

[1]Università del Piemonte Orientale, Dipartimento di Scienze e Innovazione Tecnologica-DiSIT, Vercelli, 13100, Italy;

[2]Centre of Applied Studies for the Sustainable Management and Protection of Mountain Areas (CRC Ge.S.Di.Mont.), University of Milan, Via Morino 8, 25048 Edolo (BS), Italy;

[3]Department of Agricultural and Environmental Sciences - Production, Landscape, Agroenergy (DISAA), Via Celoria 2, 20133 Milan, Italy;

[4]Water Research Institute-National Research Council (IRSA-CNR), Verbania, Italy;

[5]Università del Piemonte Orientale, Centro di Ricerca Traslazionale sulle Malattie Autoimmuni e Allergiche– CAAD, Novara, 28100, Italy

*chiara.cattaneo@uniupo.it

Fig S1. Average air temperature, rainfall and relative humidity of the year 2018, Crodo and Viganella, Italy.

Crodo			
	Avg T (°C)	Rainfall (mm)	Humidity (%)
Jan	3,6	-	83
Feb	1,4	-	67
Mar	5,3	-	71
Apr	12,8	-	63
May	15,2	191,2	79
June	19,8	71,8	66
July	22,2	69,6	65
Aug	21,2	81,8	70
Sep	18,1	23,2	72
Oct	13,1	285,2	77
Nov	6,9	283,6	89
Dec	4,4	33,2	70

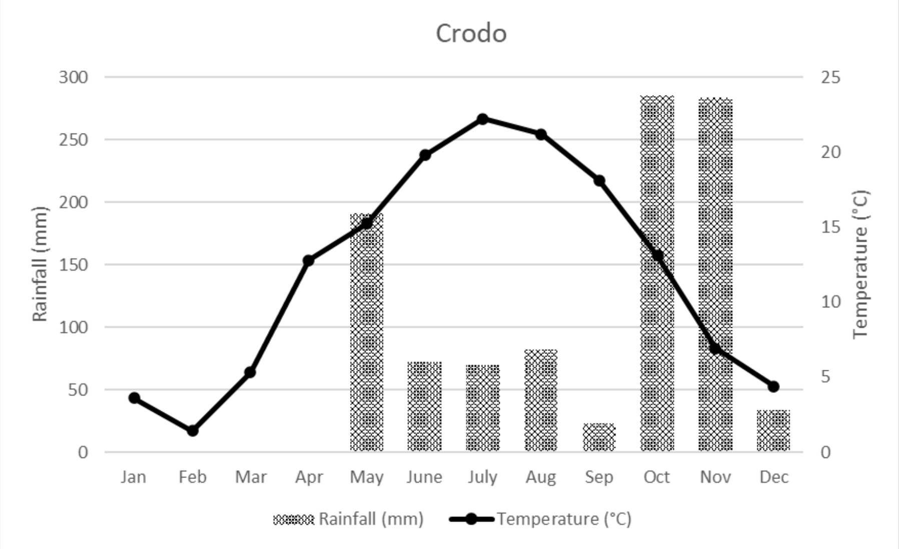

Viganella (Alpe Cheggio)			
	Avg T (°C)	Rainfall (mm)	Humidity (%)
Jan	-	-	-
Feb	-4,5	-	15
Mar	-	-	-
Apr	6	-	23
May	9,4	306,6	32
June	14,2	97,8	11
July	17	94,2	26
Aug	16,5	155,6	25
Sep	13,5	60,8	11
Oct	8,8	350,6	26
Nov	2,6	-	33
Dec	1,5	-	12

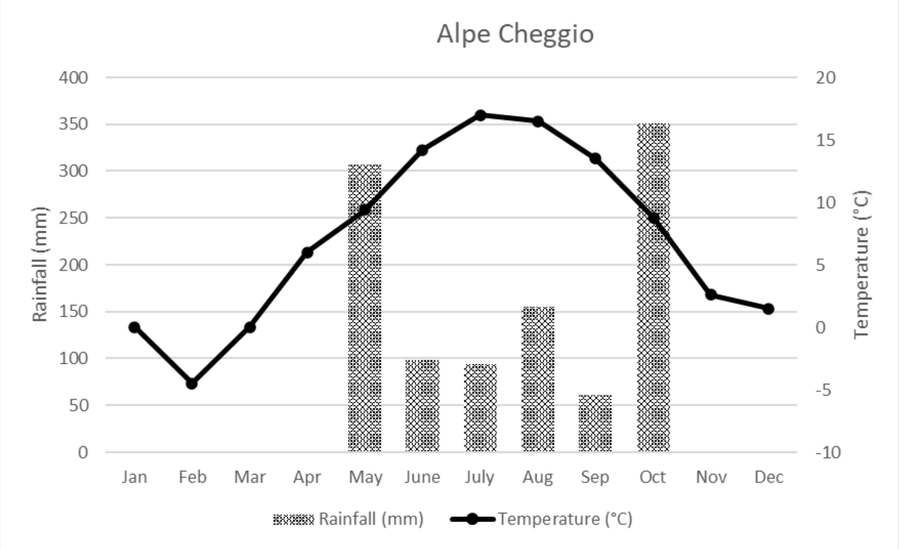

Fig S2. Box-plot representing the weight of Finola and Futura 75 seeds from different sources: certified (Cert), Crodo and Viganella.

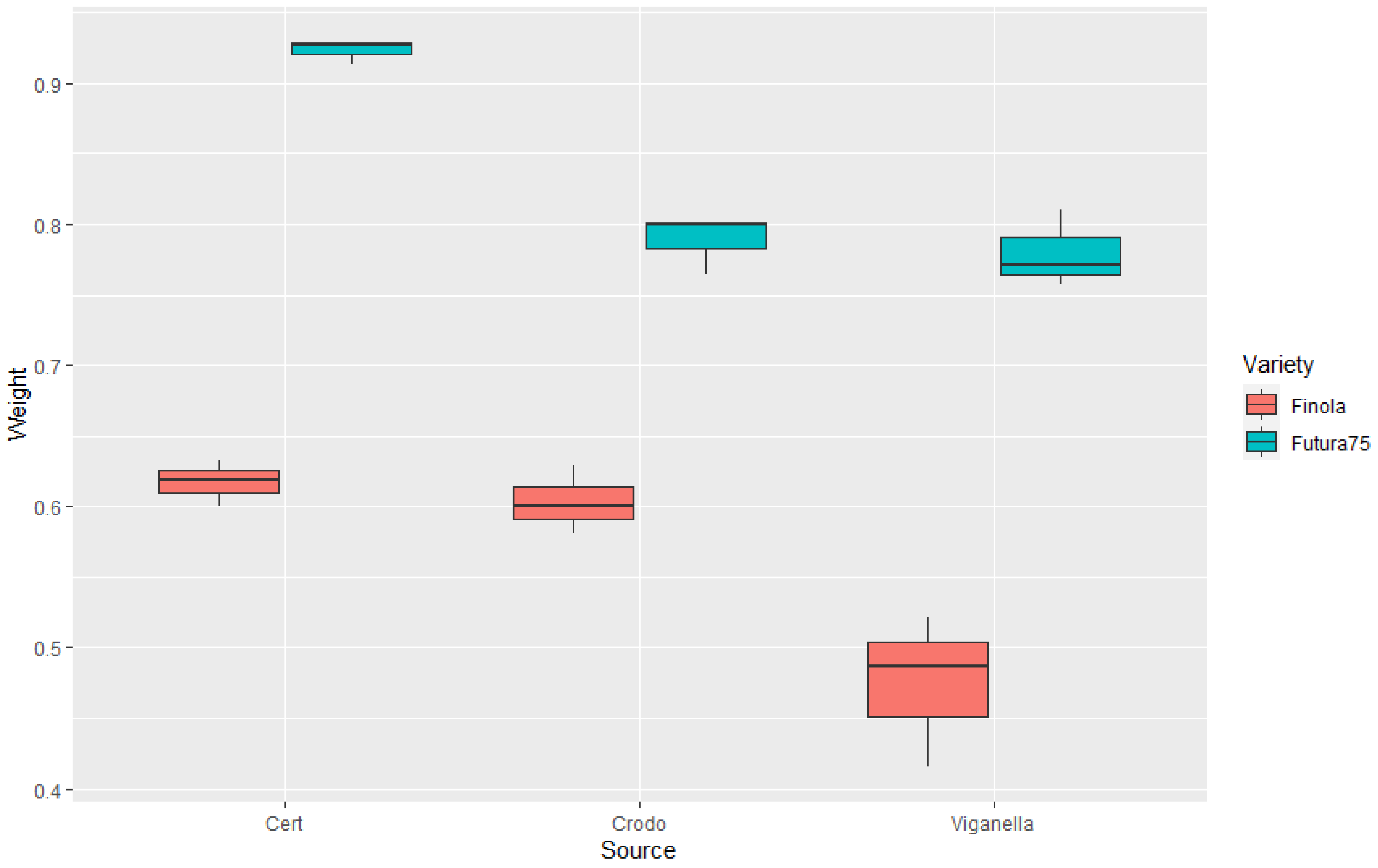

Fig S3. Total content and ratio of carotenoids and chlorophylls (µg/g) from Finola and Futura 75 certified and harvested seeds from Crodo and Viganella experimental fields. Significant different values (P<0.05, Tukey post-hoc test, Bonferroni adjustment) are indicated by different letters.

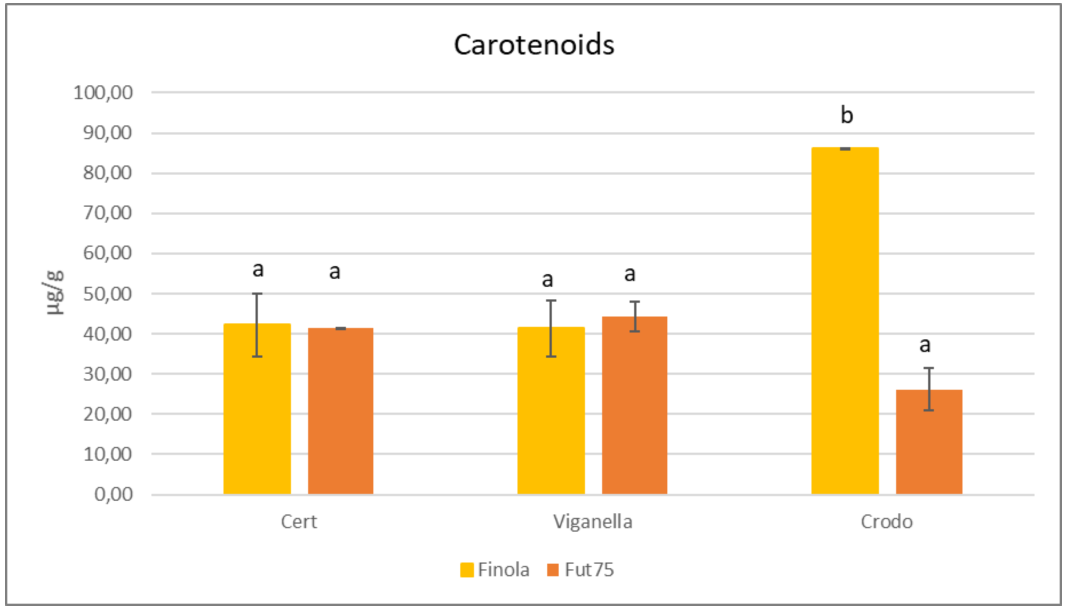

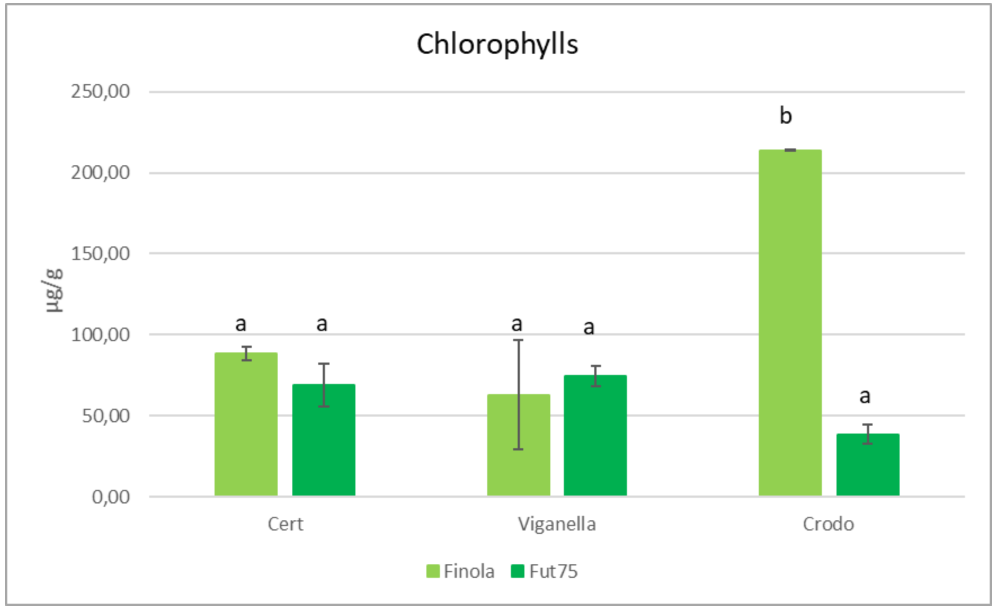

Hemp variety	Source	Car/chl ratio
Futura 75	Certified	0,60
Futura 75	Viganella	0,59
Futura 75	Crodo	0,68
Finola	Certified	0,48
Finola	Viganella	0,66
Finola	Crodo	0,40

Fig S4. SDS-PAGE of the albumin fraction of seeds from Futura 75 (1) and Finola (2) cultivars of *C. sativa* from the experimental fields of Crodo and Viganella, and certified seeds.

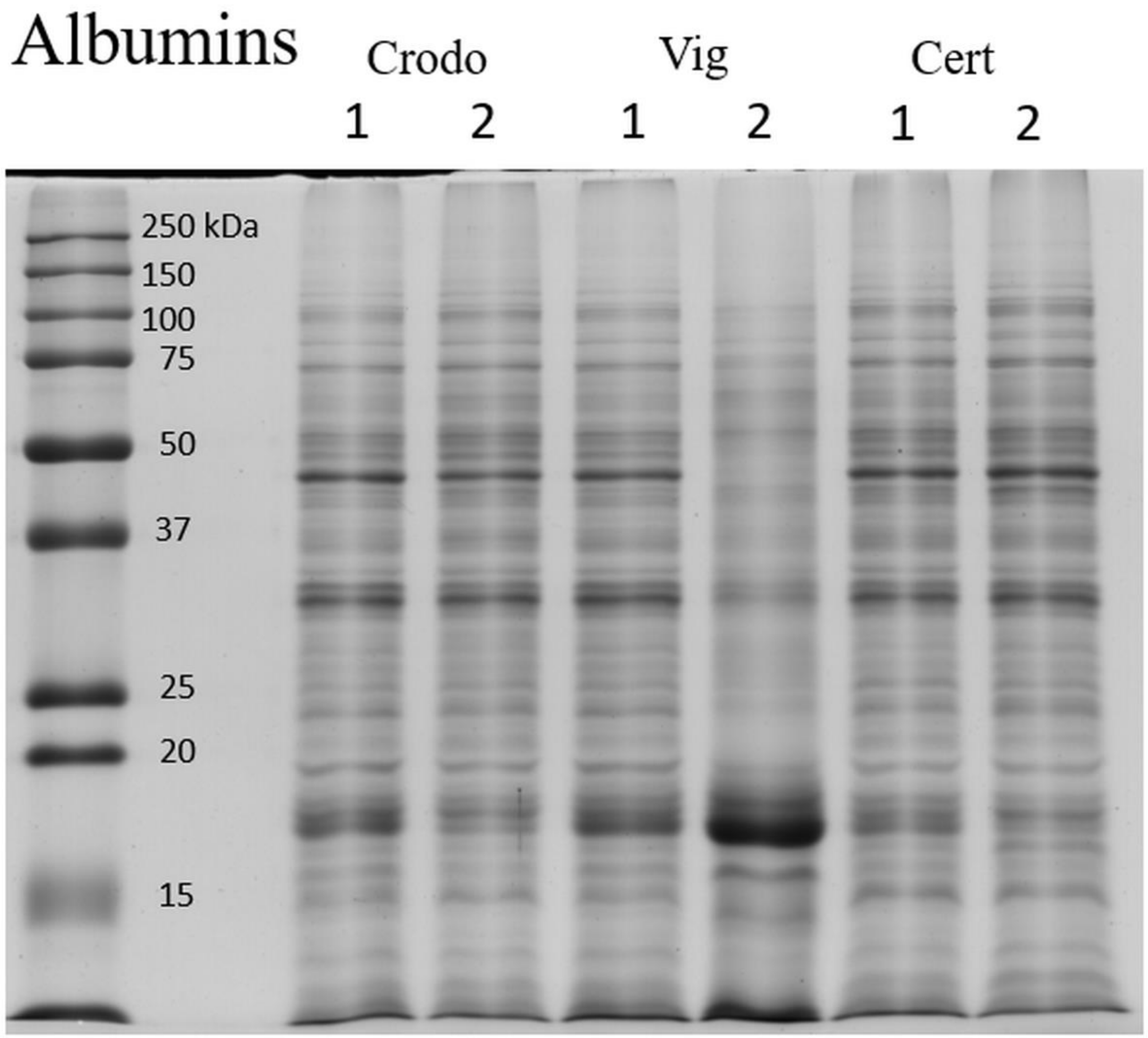

Fig S5. SDS-PAGE of the globulin fraction of seeds from Futura 75 (1) and Finola (2) cultivars of *C. sativa* from the experimental fields of Crodo and Viganella, and certified seeds.

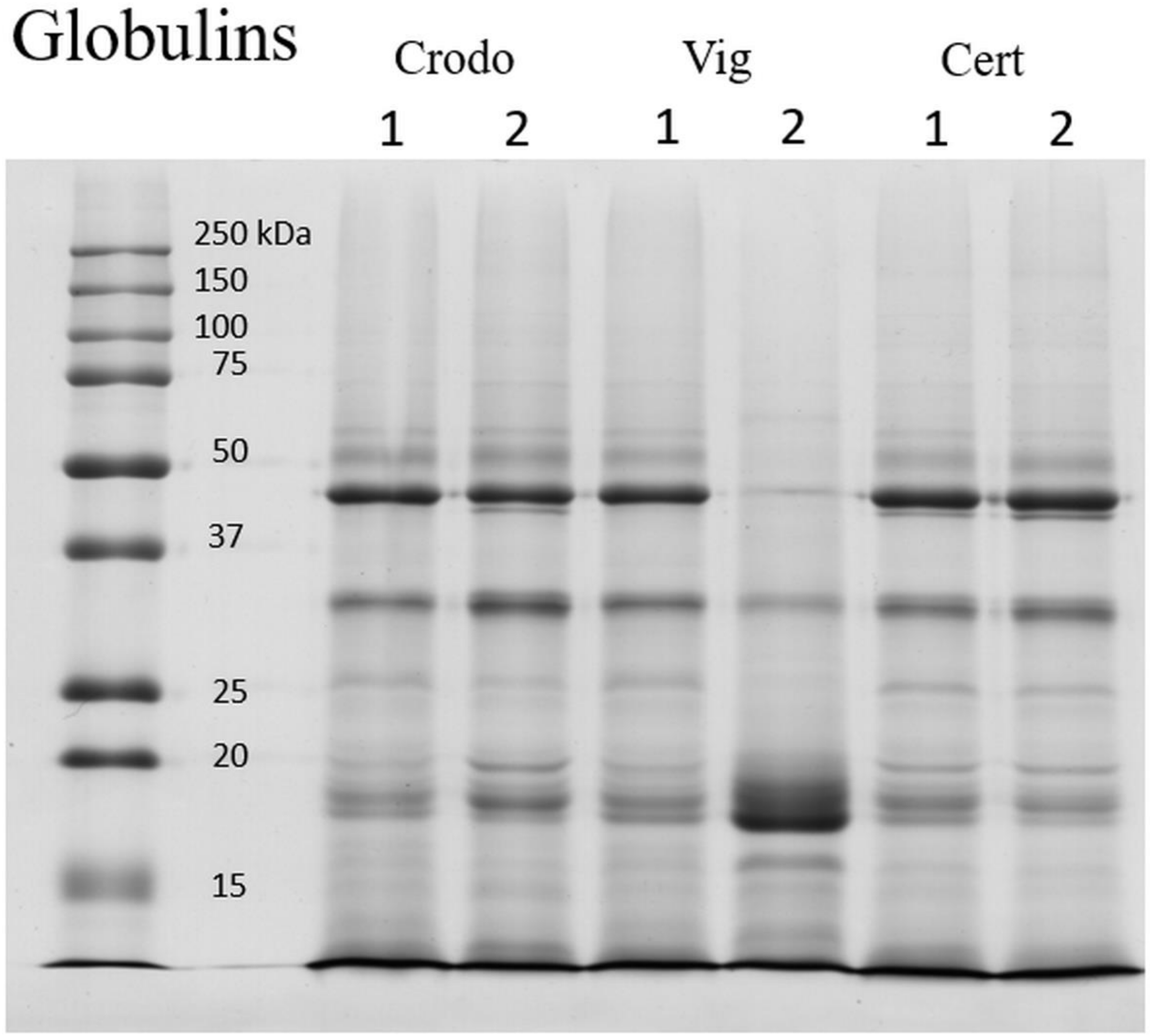

Fig S6. SDS-PAGE of the glutelin-like fraction of seeds from Futura 75 (1) and Finola (2) cultivars of *C. sativa* from the experimental fields of Crodo and Viganella, and certified seeds.

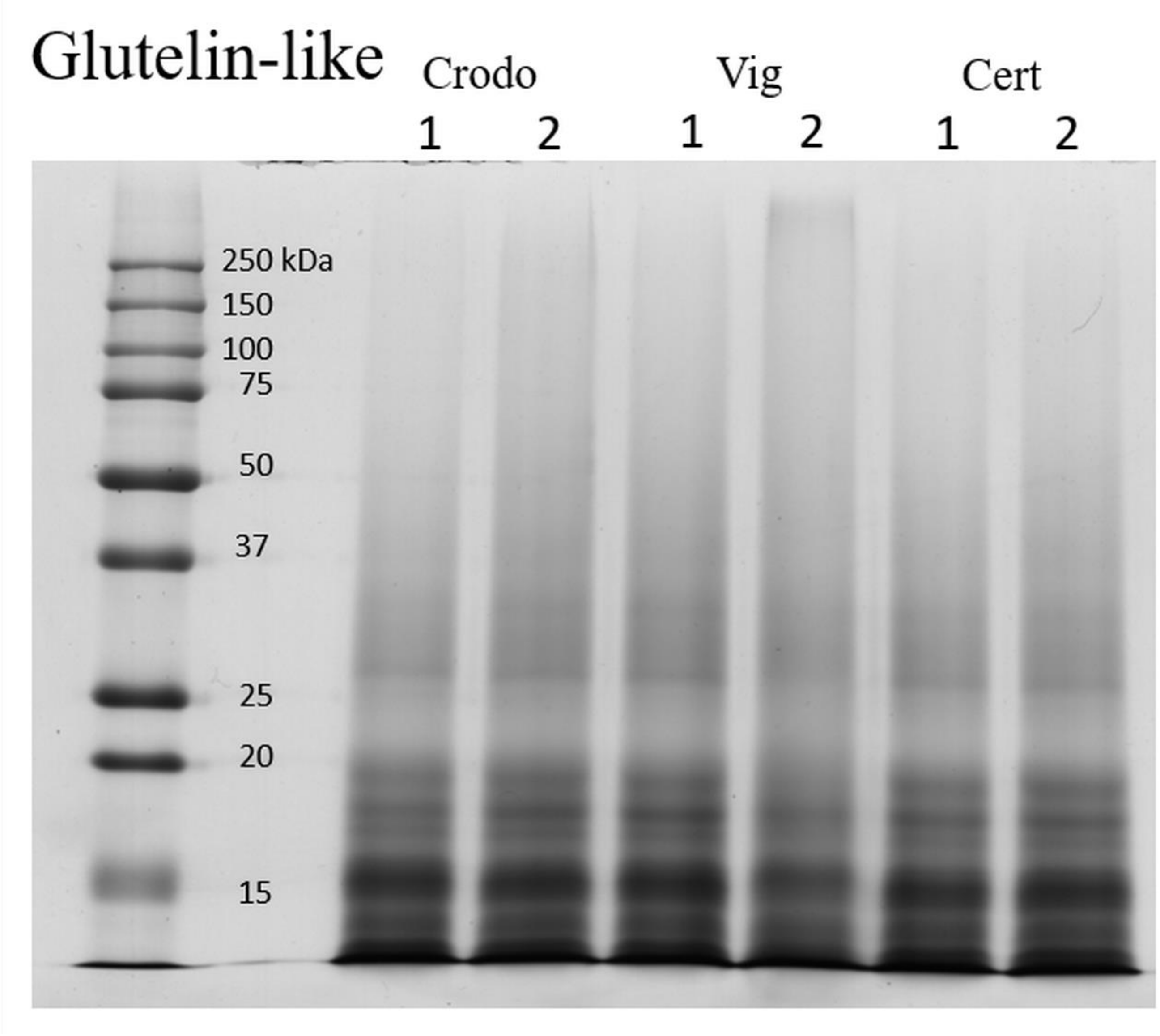

Fig S7. Representative 2D gel of total protein extracts from Finola seeds of Viganella. Spots showing variations of intensity after PDQuest image analysis are numbered.

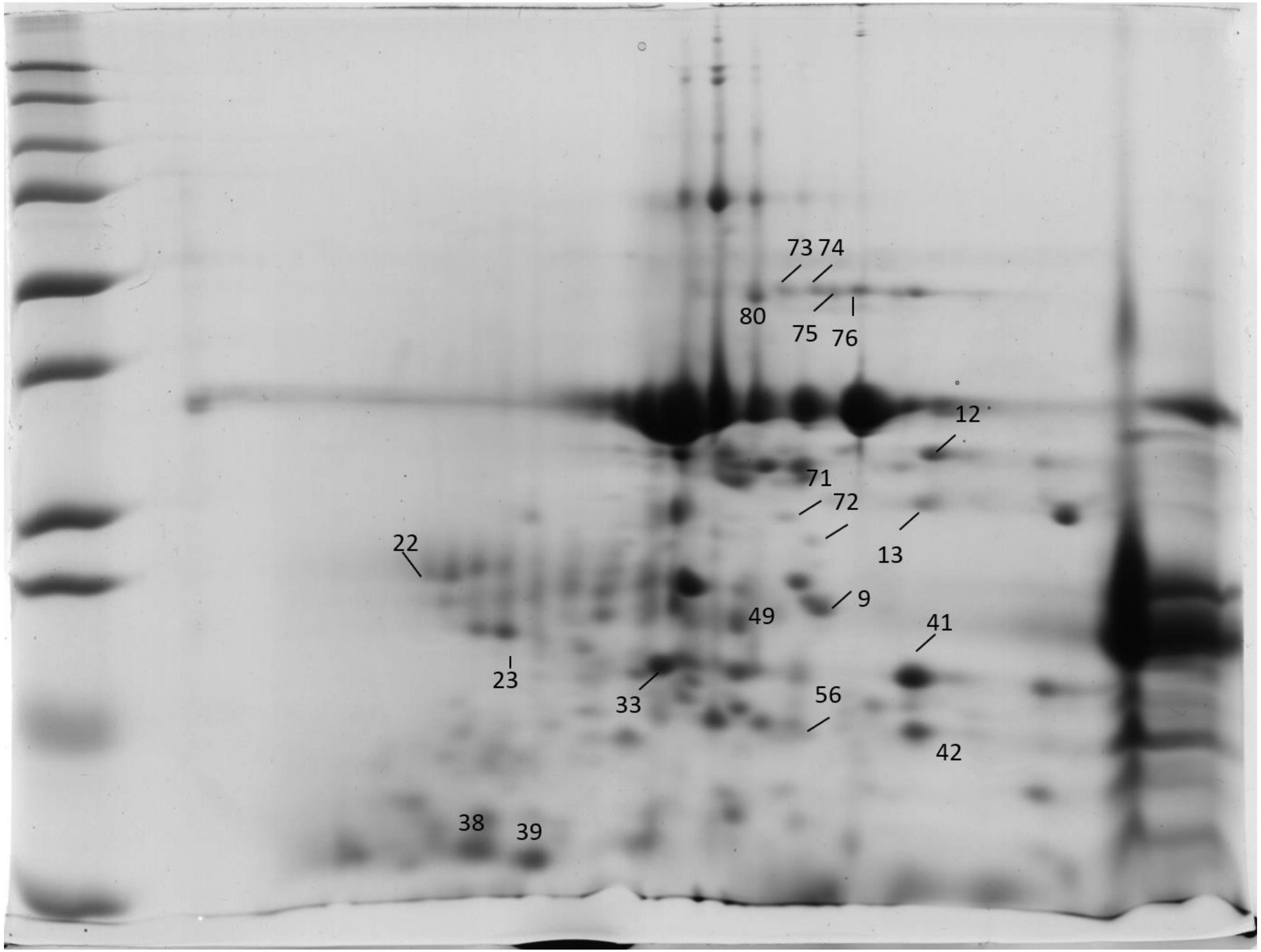

Table S1. MS/MS identifications of albumin protein extracts from hemp seeds of Futura75 and Finola analyzed by 2D-PAGE. The spot name (SP), accession code (AC), protein name and organism, number of significant matches (M) and sequences (Seq), peptide sequences, theoretical molecular mass (Mr) and database used for MS/MS search (N: NCBI, S: SwissProt) are indicated. Where available, results of blast analysis are indicated in the "Blast" column.

SP	AC	Protein name	M	Seq	Peptide sequence	Mr	DB	Blast
**1**	OAP03364.1	SRG3 (*Arabidopsis thaliana*)	1	1	R.NPALVNKIK.E + Deamidated (NQ)	40984	N	
**3**	XP_021757355.1	Phosphoglycerate kinase 3, cytosolic (*Chenopodium quinoa*)	1	1	K.MANDSVGEEVEK.L	42430	N	
**3**	GER33867.1	Phosphoglycerate kinase (*Striga asiatica*)	1	1	R.VDLNVPLDDSLKITDDTR.I	42305	N	
**3**	KAF4347106.1	Hypothetical protein G4B88_025149 (*Cannabis sativa*)	2	2	K.LAELSGK.G<br>K.GVTTIIGGGDSVAAVEK.V	92335	N	
**3**	XP_030508778.1	ADP-ribosylation factor 1 (*Cannabis sativa*)	1	1	R.DAVLLVFANK.Q	20624	N	
**4**	XP_030485298.1	Hexokinase-2, chloroplastic (*Cannabis sativa*)	1	1	K.QLSIDSGILMK.W	54139	N	
**5**	XP_030507162.1	1-Cys peroxiredoxin (*Cannabis sativa*)	1	1	K.DSSGNQVPSR.A	24231	N	
**7**	LGUL_ORYSJ	Lactoylglutathione lyase (*Oryza sativa subsp. japonica*)	1	1	R.QPGPLPGLNTK	32875	S	

**8**	CDP79027.1	Edestin 2 (*Cannabis sativa*)	6	3	R.GEDLQIIAPSR.I<br>R.SEGASSDEQHQK.V<br>R.ESGEQTPNGNIFSGFDTR.I + Deamidated (NQ)	56338	N	
**8**	XP_030506286.1	NADPH-dependent aldehyde reductase 1, chloroplastic-like (*Cannabis sativa*)	1	1	K.IALVSGGDSGIGR.A	32167	N	
**11**	RVX02887.1	putative ribonuclease H protein (*Vitis vinifera*)	1	1	R.ALWELVLGL.-	37231	N	
**13**	XP_030504301.1<br>XP_030506109.1	18.5 kDa class I heat shock protein (*Cannabis sativa*)	4	2	K.LSEENSAFVNAR.V<br>K.AAMENGVLTVTVPK + Oxidation (M)	18013<br>17999	N	
**14**	XP_030503293.1	18.5 kDa class I heat shock protein-like (*Cannabis sativa*)	19	6	R.SNILDPFSMDVWDPFKDFPLSVPDISK.E + Oxidation (M)<br>K.DFPLSVPDISK.E<br>K.ETSAMVNAR.V + Oxidation (M)<br>K.ADVPGLK.K<br>R.VLQISGER.N<br>K.AAMENGVLTVTVPK.E + Deamidated (NQ), Oxidation M	17486	N	
**15**	XP_030485488.1<br>XP_030488139.1	18.1 kDa class I heat shock protein-like (*Cannabis sativa*)	1	1	K.ASMENGVLTVVVPK.E + Deamidated (NQ), Oxidation M	17886/<br>17852	N	
**16**	HSP11_MEDSA	18.1 kDa class I heat shock protein (Fragment) (*Medicago sativa*)	2	2	R.VLQISGER.N<br>K.AAMENGVLTVTVPK.E + Deamidated (NQ), Oxidation M	17486	S	XP_030503293.1 18.5 kDa class I heat shock protein-like (*Cannabis sativa*)

**16**	TKS00882.1	NADH dehydrogenase (*Populus alba*)	1	1	R.VEAAMVNAR.I + Oxidation (M)	81298	N	
**17**	XP_030499617.1	Glycine-rich RNA-binding protein-like (*Cannabis sativa*)	1	1	R.DAIEGMNGQDLDGR.N + Deamidated (NQ); Oxidation (M)	17128	N	
**19**	CDP79023.1	Edestin 1 (*Cannabis sativa*)	1	1	R.GQGQGQSQGSQPDR.H	58810	N	
**20**	CXXS1_ARATH	Thioredoxin-like protein CXXS1 *Arabidopsis thaliana*	1	1	K.LVGANPDEIK.K + Deamidated (NQ)	13440	S	KAF4398011.1 hypothetical protein G4B88_019732 (*Cannabis sativa*), XP_030491271.1 thioredoxin-like protein CXXS1 (*Cannabis sativa*)
**23**	CXXS1_ARATH	Thioredoxin-like protein CXXS1 *Arabidopsis thaliana*	2	1	K.LVGANPDEIK.K + Deamidated (NQ)	13440	S	KAF4398011.1 hypothetical protein G4B88_019732 (*Cannabis sativa*), XP_030491271.1 thioredoxin-like protein CXXS1 (*Cannabis sativa*)
**25**	XP_030506286.1	NADPH-dependent aldehyde reductase 1, chloroplastic-like (*Cannabis sativa*)	2	1	K.IALVSGGDSGIGR.A	32167	N	
**27**	CAB00000.1	ribulose-1,5-bisphosphate carboxylase/oxygenase large subunit, partial (chloroplast) (*Caryocar glabrum*)	1	1	K.DTDILAAFR.V	50042	N	

**27**	CDP79023.1	edestin 1 (*Cannabis sativa*)	1	1	R.GQGQGQSQGSQPDRHQK.L + 2 Deamidated (NQ)	58810	N	
**28**	CXXS1_ARATH	Thioredoxin-like protein CXXS1 *Arabidopsis thaliana*	2	1	K.LVGANPDEIK.K + Deamidated (NQ)	13440	S	KAF4398011.1 hypothetical protein G4B88_019732 (*Cannabis sativa*), XP_030491271.1 thioredoxin-like protein CXXS1 (*Cannabis sativa*)
**29**	ABX09991.1	actin 1, partial (*Ziziphus jujuba*)	3	1	K.LCYVALDFEQEMATAASSSSLEK.S K.LCYVALDFEQEMATAASSSSLEK.S + Deamidated (NQ)K.LCYVALDFEQEMATAASSSSLEK.S + Oxidation (M)	17833	N	
**29**	CDP79023.1	edestin 1 (*Cannabis sativa*)	1	1	R.GQGQGQSQGSQPDR.H	58810	N	
**29**	PNY00005.1	Tubulin alpha-3 chain-like protein (*Trifolium pratense*)	1	1	R.FDGAINVDITEFQTNLVPYPR.I	50123	N	
**29**	AGJ50594.1	beta-tubulin (*Pericallis cruenta*)	2	2	K.NSSYFVEWIPNNVK.S + Deamidated (NQ) K.GHYTEGAELVDSVLDVVR.K	50697	N	
**29**	XP_023875501.1	GTP-binding protein rhoA (*Quercus suber*)	1	1	K.TCLLIVFSK.G	21978	N	
**32**	XP_030478962.1	Heat shock 70 kDa protein-like (*Cannabis sativa*)	2	2	R.FSDPSVQSDMK.L + Oxidation (M) R.TTPSYVAFTDTER.L	72426	N	

**32**	XP_030501851.1<br>XP_030492979.1	Luminal-binding protein 5 isoform X1 (*Cannabis sativa*) and mediator of RNA polymerase II transcription subunit (*Cannabis sativa*)	1	1	K.DAGTIAGLNVAR.I	73647<br>73541	N
**33**	RUBA_RICCO	RuBisCO large subunit-binding protein subunit alpha (Fragment) (*Ricinus communis)*	2	2	R.VLVTDQK.I<br>K.TNDSAGDGTTTASVLAR.E	52461	S
**33**	PDI11_ARATH	Protein disulfide isomerase-like 1-1 (*Arabidopsis thaliana*)	1	1	K.QSGPASAEIK.S	55852	S
**38**	XP_030508280.1<br>XP_030508281.1	vicilin C72-like (*Cannabis sativa*)	1	1	R.ADVIVVPAGSTVYMTNQDNK.E + Oxidation (M)	99860<br>84760	N
**47**	HSP11_DAUCA	17.8 kDa class I heat shock protein (*Daucus carota*)	1	1	R.VLQISGER.N	17771	S
**53**	XP_030507162.1	1-Cys peroxiredoxin (*Cannabis sativa*)	1	1	K.DSSGNQVPSR.A	24231	N
**53**	XP_030486208.1	glutathione S-transferase DHAR2-like (*Cannabis sativa*)	1	1	K.VSAVDLSLAPK.L	23764	N
**54**	XP_030482081.1	11 kDa late embryogenesis abundant protein (*Cannabis sativa)*	6	2	K.ESAANVAASAK.S<br>R.TTTQDPSGGAPGYGTGGY.-	15573	N
**54**	SNQ45158.1	Edestin 3 (*Cannabis sativa*)	6	4	R.AKVNQLAGK.V<br>R.ADVFSPQAGR.L<br>R.AMPEDVIANSYQISR.E<br>R.QGQALTVPQNFAVVK.M	56672	N
**54**	SNQ45160.1	Edestin 3 (*Cannabis sativa*)	7	5	R.ENMGDPAR.A + Oxidation (M)<br>R.AkVNQLAGK.V<br>R.ADVFSPQAGR.L	56473	N

					R.AMPEDVIANSYQISR.E<br>R.QGQALTVPQNFAIVK.M		
**54**	CDP79023.1	Edestin 1 (*Cannabis sativa*)	1	1	K.TNDNAWVSPLAGR.T	58810	N
**54**	CDP79027.1	Edestin 2 (*Cannabis sativa*)	3	1	R.LQVVDDNGR.N + Deamidated (NQ)	56338	N
**54**	CP19D_ARATH	Peptidyl-prolyl cis-trans isomerase CYP19-4 (*Arabidopsis thaliana*)	2	1	K.VVIADSGELPL.-	21577	S
**55**	SNQ45160.1	Edestin 3 (*Cannabis sativa*)	32	4	R.ADVFSPQAGR.L<br>R.QGQALTVPQNFAIVK.M<br>K.MAENEGFEWISFK.T + Oxidation (M) R.AMPEDVIANSYQISR.E	56473	N
**55**	SNQ45158.1	Edestin 3 (*Cannabis sativa*)	30	3	R.ADVFSPQAGR.L<br>R.AMPEDVIANSYQISR.E<br>R.QGQALTVPQNFAVVK.M	56080	N
**55**	CDP79023.1	Edestin 1 (*Cannabis sativa*)	23	6	R.ENIGDPSR.A<br>R.ENIGDPSRADVFTPQAGR.I<br>R.ISTVNSYNLPILR.F<br>R.VQVVNHMGQK.C + Oxidation (M)<br>K.QASSDGFEWVSFK.T<br>K.TNDNAWVSPLAGR.T	58810	N
**55**	CDP79028.1	Edestin 2 (*Cannabis sativa*)	26	7	R.LNTLNNYNLPILR.F<br>R.LQVVDDNGR.N<br>R.LQVVDDNGRNVFDGELR.E + Deamidated (NQ)<br>K.ASAQGFEWIAVK.T<br>K.TNDNAMRNPLAGK.V + Oxidation (M)<br>R.AMPDDVLANAFQISR.E<br>R.DEISVFSPSSQQTR.Y	56277	N

**55**	XP_030494449.1	60S ribosomal protein L12-3-like (*Cannabis sativa*)	3	3	R.VTGGEVGAASSLAPK.I<br>K.VSVVPSAAALVIK.A<br>K.DLQEEISDGDVEIPQD.-	17893	N
**56**	SNQ45153.2<br>XP_030504501.1	7S vicilin-like protein (*Cannabis sativa*)	1	1	R.IGFITMEPK.T + Oxidation (M)	56026	N
**56**	XP_030487452.1	Triosephosphate isomerase, chloroplastic (*Cannabis sativa*)	1	1	K.NNVSAEVASK.I + Deamidated (NQ)	34234	N
**57**	SNQ45153.2<br>XP_030504501.1	7S vicilin-like protein (*Cannabis sativa*)	2	1	R.IGFITMEPK.T + Oxidation (M)	56026	N
**58**	CDP79023.1,<br>CDP79024.1,<br>CDP79026.1	Edestin 1 (*Cannabis sativa*)	1	1	R.GQGQGQSQGSQPDR.H	58810,<br>58829,<br>58740	N
**59**	XP_030479619.1	Late embryogenesis abundant protein, group 3-like (*Cannabis sativa*)	3	3	K.DAITGDGK.-<br>K.ETAQDLSDSAK.G<br>K.QDAGFNTDDITK.R	20590	N
**65**	OVA13829.1	Ubiquitin-associated domain/translation elongation factor EF-Ts (*Macleaya cordata*)	1	1	K.DIELVMTQAGVPR.S + Oxidation (M)	22359	N
**A**	CDP79023.1,<br>CDP79024.1,<br>CDP79026.1	Edestin 1 (*Cannabis sativa*)	1	1	R.GQGQGQSQGSQPDR.H	58810,<br>58829,<br>58740	N
**B**	CDP79023.1,<br>CDP79024.1,<br>CDP79026.1	Edestin 1 (*Cannabis sativa*)	2	1	R.GQGQGQSQGSQPDR.H	58810,<br>58829,<br>58740	N
**C**	CDP79023.1,<br>CDP79024.1,<br>CDP79026.1	Edestin 1 (*Cannabis sativa*)	6	2	R.GQGQGQSQGSQPDR.H<br>R.GQGQGQSQGSQPDRHQK.L	58810,<br>58829,<br>58740	N
**C**	OAP03364.1		2	1	R.NPALVNKlK.E + Deamidated (NQ)	40984	N

**D**	CDP79023.1, CDP79024.1, CDP79026.1	Edestin 1 (*Cannabis sativa*)	6	2	R.GQGQGQSQGSQPDR.H<br>R.GQGQGQSQGSQPDRHQK.L	58810, 58829, 58740	N
**E**	CDP79023.1, CDP79024.1, CDP79026.1	Edestin 1 (*Cannabis sativa*)	9	5	R.QQNQCQIDR.I + Deamidated (NQ)<br>R.GQGQGQSQGSQPDR.H<br>R.GQGQGQSQGSQPDRHQK.L<br>R.FYLAGNPEDEFEQLR.R<br>R.YTIQQNGLHLPSYTNTPQLVYIVK.G + Deamidated (NQ)	58810, 58829, 58740	N
**F**	SNQ45158.1	Edestin 3 (*Cannabis sativa*)	6	2	R.GVLGTLFPGCAETFEEAQVSVGGGR.S<br>R.LTIQPNGLHLPSYTNGPQLIHVIR.G + Deamidated (NQ)	56672	N
**F**	CDP79023.1, CDP79024.1, CDP79026.1	Edestin 1 (*Cannabis sativa*)	5	4	R.GQGQGQSQGSQPDR.H<br>R.FYLAGNPEDEFEQLR.R<br>R.FYLAGNPEDEFEQLRR.E<br>R.YTIQQNGLHLPSYTNTPQLVYIVK.G + Deamidated (NQ)	58810, 58829, 58740	N
**F**	VAH03463.1<br>HSP7F_ARATH	Unnamed protein product (*Triticum turgidum* subsp. Durum)<br>Heat shock 70 kDa protein 6, chloroplastic (*Arabidopsis thaliana*)	1	1	K.VTKAVITVPAYFNDSQR.T + Deamidated (NQ)	27644, 76575	N<br>S
**F**	XP_004290029.1	PREDICTED: chaperonin CPN60-2, mitochondrial-like (*Fragaria vesca* subsp. Vesca)	1	1	R.TALVDAASVSSLMTTTEAVVVSLPEK.E	61759	N
**G**	CDP79023.1, CDP79024.1, CDP79026.1	Edestin 1 (*Cannabis sativa*)	1	1	R.GQGQGQSQGSQPDR.H	58810, 58829, 58740	N
**H**	SNQ45158.1	Edestin 3 (*Cannabis sativa*)	1	1	R.LTIQPNGLHLPSYTNGPQLIHVIR.G + 3 Deamidated (NQ)	56672	N

**I**	KZN00901.1	Hypothetical protein DCAR_009655 (*Daucus carota* subsp. Sativus)	1	1	R.VPTANVSVVDLTCR.L	36278	N
**L**	CDP79023.1, CDP79024.1, CDP79026.1	Edestin 1 (*Cannabis sativa*)	2	1	R.GQGQGQSQGSQPDR.H	58810, 58829, 58740	N
**M**	CDP79023.1, CDP79024.1, CDP79026.1	Edestin 1 (*Cannabis sativa*)	3	2	R.GQGQGQSQGSQPDRHQK.L<br>R.GQGQGQSQGSQPDR.H	58810, 58829, 58740	N
**M**	EMS59070.1	Histone H4 (*Triticum urartu*)	2	2	R.ISGLIYQETR.G + Deamidated (NQ)<br>K.TVTSMDVVYALK.R + Oxidation (M)	11418	N
**N**	ARF_MAIZE	ADP-ribosylation factor 1 (*Cannabis sativa*)	1	1	R.DAVLLVFANK.Q	20705	S
**N**	CDP79023.1, CDP79024.1, CDP79026.1	Edestin 1 (*Cannabis sativa*)	2	1	R.GQGQGQSQGSQPDR.H	58810, 58829, 58740	N
**N**	KZN00901.1	Hypothetical protein DCAR_009655 (*Daucus carota* subsp. Sativus)	1	1	R.VPTANVSVVDLTCR.L	36278	N
**N**	ABX09991.1	Actin 1, partial (*Ziziphus jujuba*)	2	1	K.LCYVALDFEQEMATAASSSSLEK.S	17833	N
**N**	XP_022159030.1	Uncharacterized protein LOC111025474 (*Momordica charantia*)	1	1	K.RAAEAKSVEEAEPR.G	31380	N
**N**	XP_023875501.1	GTP-binding protein rhoA (*Quercus suber*)	1	1	K.TCLLIVFSK.G	21978	N
**R**	CDP79027.1	Edestin 2 (*Cannabis sativa*)	2	1	R.GLLLPSFLNAPMMFYVIQGR.G + 2 Oxidation (M)	56338	N

Table S2. MS/MS identifications of total protein extracts from hemp seeds of Futura75 and Finola analyzed by SDS-PAGE. Band identificative name (ID), accession code (AC), protein name and organism, number of significant matches (M) and sequences (Seq), peptide sequences and theoretical molecular mass (Mr) are indicated.

ID	AC	Description name	M	Seq.	Peptide sequence	Mr
**A1**	CDP79023.1	edestin 1 (*Cannabis sativa*)	3	3	K.TNDNAWVSPLAGR.T<br>R.EETVLLTSSTSSR.R<br>R.GQGQGQSQGSQPDR.H	58810
**A1**	XP_030508280.1	vicilin C72-like (*Cannabis sativa*)	1	1	R.ADVIVVPAGSTVYMTNQDNK.E + Oxidation (M)	99860
**A2**	CDP79023.1	edestin 1 (*Cannabis sativa*)	2	2	R.GQGQGQSQGSQPDR.H + Deamidated (NQ)<br>R.YLEEAFNVDSETVK.R + Deamidated (NQ)	58810
**A3**	CDP79023.1	edestin 1 (*Cannabis sativa*)	1	1	R.GQGQGQSQGSQPDR.H	58810
**A4**	CDP79023.1	edestin 1 (*Cannabis sativa*)	1	1	R.GQGQGQSQGSQPDR.H	58810
**A5**	CDP79023.1	edestin 1 (*Cannabis sativa*)	1	1	R.GQGQGQSQGSQPDR.H	58810
**A6**	CDP79023.1	edestin 1 (*Cannabis sativa*)	19	11	R.LQGQNDDR + Deamidated (NQ) ;<br>K.GTLDLVSPLR.S;<br>R.QQNQCQIDR.I ;<br>K.TNDNAWVSPLAGR.T ;<br>R.EETVLLTSSTSSR.R;	58810

					R.GQGQGQSQGSQPDR.H ;<br>R.LQGQNDDRNSIIR.V + Deamidated (NQ) ;<br>R.YLEEAFNVDSETVK.R;<br>R.FYLAGNPEDEFEQLR.R;<br>R.GILGVTFPGCPETFEESQR.G	
**A6**	XP_030478962.1	Heat shock 70 kDa protein-like (*Cannabis sativa*)	1	1	R.TTPSYVAFTDTER.L<br>K.DAGAISGLNVLR.I	72426
**A6**	CDP79027.1	Edestin 2 (*Cannabis sativa*)	1	1	R.DEISVFSPSSQQTR.Y	56338
**A7**	XP_030508280.1	Vicilin C72-like (*Cannabis sativa*)	48	10	R.TVGFGVNAR.N ;<br>K.LSYFVSQQQQEEGR.G + Deamidated (NQ) ;<br>K.FYEVTPEQNK.Q ;<br>K.TTVLVMVVEGTGR.M ;<br>K.LSYFVSQQQQEEGR.G ;<br>R.GHESSGPVISLQNQSPR.Y ;<br>R.ADVIVVPAGSTVYMTNQDNK.E ;<br>K.EAQELAFNMQGSEVEQIFNQPK.L ;<br>R.VTAQLSPGDVFIIPAGHPVAVVANNNQK.L ;<br>R.LAVLEAQPSTFVAPHHCDADSVLVVTK.G	99860
**A7**	XP_030493178.1	Sucrose-binding protein-like (*Cannabis sativa*)	15	6	K.ELAFSVPAR.E ;<br>R.SSGPFNLFR.D ;<br>K.LINPVSLPGR.F ;<br>K.SQNEEYFFPGPR.S ;<br>R.FEPFYGAGGENPESFYK.A ;<br>R.ETFNLVEGDILNIPAGTPVYIVNR.D	55771
**A7**	CDP79023.1	Edestin 1 (*Cannabis sativa*)	7	5	K.TNDNAWVSPLAGR.T ;<br>R.GQGQGQSQGSQPDR.H ;<br>K.QASSDGFEWVSFK.T ;	58810

					R. YLEEAFNVDSETVK.R + 2 Deamidated (NQ) ; R.GILGVTFPGCPETFEESQR.G	
**A7**	AAB01374.1	Beta-conglycinin storage protein (*Glycine max*)	2	2	R.LQESVIVEISK ; R.FESFFLSSTQAQQSYLQGFSK.N	74565
**A8**	CDP79028.1	Edestin 2 (*Cannabis sativa*)	66	15	R.QNIDRPSQADIFNPR.G ; R.GEDLQIIAPSR.I ; R.WQSQCQFQR.L ; R.SEGASSDEQHQK.V ; R.VRGEDLQIIAPSR.I ; R.LNTLNNYNLPILR.F ; R.LNTLNNYNLPILR.F + Deamidated (NQ) ; R.DEISVFSPSSQQTR.Y ; R.AMPDDVLANAFQISR.E + Oxidation (M) ; R.ILAESFNVDTELAHK.L ; R.GIHGAVIPGCPETFER.G ; R.ESGEQTPNGNIFSGFDTR.I ; R.GLLLPSFLNAPMMFYVIQGR.G + 2 Oxidation (M) ; R.VECEAGVSEYWDIQNTEDDELHCAGVETAR.H	56277
**A8**	CDP79023.1	Edestin 1 (*Cannabis sativa*)	20	11	R.QQNQCQIDR.I ; R.FYLAGNPEDEFEQLR.R ; K.TNDNAWVSPLAGR.T ; R.EETVLLTSSTSSR.R ; R.GQGQGQSQGSQPDR.H ; K.QASSDGFEWVSFK.T ; R.ISTVNSYNLPILR.F ; R.YLEEAFNVDSETVK.R ; R.FYLAGNPEDEFEQLR.R ; R.GILGVTFPGCPETFEESQR.G	58810
**A8**	SNQ45160.1	Edestin 3 (*Cannabis sativa*)	13	7	K.QQEGLPNNVFR.G ; R.GFSVNLIQEAFNVDSETAR.K ; R.TAVYGDQNECQLNR.L ; R.AMPEDVIANSYQISR.E + Oxidation (M) ;	56473

**A8**	XP_004232705.1	Lactoylglutathione lyase GLX1 (*Solanum lycopersicum*)	3	3	R.IEEEKQQEGLPNNVFR.G ; R.GVLGTLFPGCAETFEEAQVSVGGGR.S K.ITSFLDPDGWK.T ; K.DPDGYLFEIIQR.E ; K.GNAYAQIAIGTDDVYK.S	32972
**A8**	XP_030508280.1	Vicilin C72-like (*Cannabis sativa*)	1	1	R.ADVIVVPAGSTVYMTNQDNK.E + Oxidation (M)	99860
**A8**	XP_030493178.1	Sucrose-binding protein-like (*Cannabis sativa*)	1	1	R.ETFNLVEGDILNIPAGTPVYIVNR.D	55771
**A9**	SNQ45160.1	Edestin 3 (*Cannabis sativa*)	168	13	K.IQSQDDFR.G ; R.KIQSQDDFR.G ; K.QQEGLPNNVFR.G ; R.VECEGGMIESWNPNHEQFQCAGVALLR.L + Oxidation (M) ; R.QGQALTVPQNFAIVK.M ; R.TAVYGDQNECQLNR.L ;; R.AMPEDVIANSYQISR.E + Oxidation (M) ; R.FYIAGNPHEDFPQSR.R ; R.IEEEKQQEGLPNNVFR.G ; R.GFSVNLIQEAFNVDSETAR.K ; R.GVLGTLFPGCAETFEEAQVSVGGGR.S ; R.LTIQPNGLHLPSYTNGPQLIHVIR.G + 2 Deamidated (NQ) ; K.EGDIIAIPAGMAYWCNNDGDQPLVTVNLIDVSNHNNQLDLTPR.R	56473
**A9**	CDP79023.1	Edestin 1 (*Cannabis sativa*)	164	20	R.GQGQGQSQGSQPDR.H ; K.GTLDLVSPLR.S ; R.QQNQCQIDR.I ; R.VKGTLDLVSPLR.S ; K.TNDNAWVSPLAGR.T ; K.QASSDGFEWVSFK.T ;	58810

					R.ISTVNSYNLPILR.F ; R.LQGQNDDRNSIIR.V ; R.ALPEAVLANAFQISR.D ; R.YLEEAFNVDSETVKR.L ; R.FYLAGNPEDEFEQLR.R ; R.YTIQQNGLHLPSYTNTPQLVYIVK.G ; R.GILGVTFPGCPETFEESQR.G ; R.EGDIVAIPAGVAYWSYNNGDQQLVFVSLLDTSNVNNQLDDNPR.R ; R.VEAEAGLIESWNPNHNQFQCAGVAVVR.Y ; R.GILGVTFPGCPETFEESQRGQGQGQSQGSQPDR.H + Deamidated (NQ)	
**A9**	CDP79028.1	Edestin 2 (*Cannabis sativa*)	107	15	R.FLQLTAER.G; R.GEDLQIIAPSR.I; R.WQSQCQFQR.L; R.SEGASSDEQHQK.V; R.ESGEQTPNGNIFSGFDTR.I + 2 Deamidated (NQ); R.VRGEDLQIIAPSR.I; R.LNTLNNYNLPILR.F; R.AMPDDVLANAFQISR.E + Oxidation (M); R.ILAESFNVDTELAHK.L; R.GIHGAVIPGCPETFER.G; R.ESGEQTPNGNIFSGFDTR.I; R.GLLLPSFLNAPMMFYVIQGR.G + 2 Oxidation (M); R.VECEAGVSEYWDIQNTEDDELHCAGVETAR.H; K.EGDMVAMPAGVADWVYNNGDSPLVLIAFVDVGNQANQLDQFSR.R + Deamidated (NQ); 2 Oxidation (M)	56277
**A9**	XP_030506286.1	NADPH-dependent aldehyde reductase 1, chloroplastic-like (*Cannabis sativa*)	8	3	K.IALVSGGDSGIGR.A; R.VNGVAPGPIWTPLIPSSFSEDHTAK.F + Deamidated (NQ); R.IDILINNAAEQYVTSSIEEIDEAR.L	32167

**A9**	XP_030477762.1	Aspartic proteinase A1-like (*Cannabis sativa*)	2	2	K.NGKPAAIQYGTGAIAGFFSEDNVK.V + Deamidated (NQ); K.AIVAQYGETIIESLTK.D	56422
**A9**	EEF43857.1	Lactoylglutathione lyase, putative (*Ricinus communis*)	5	3	R.QPGPIPGLNTK ; K.ITSFLDPDGWK.T; K.DPDGYIFEIIQR	31641
**A9**	XP_030495935.1	LOW QUALITY PROTEIN: 3-oxoacyl-(acyl-carrier-protein) reductase 4-like (*Cannabis sativa*)	2	2	K.NLEGPVVVVTGASR.G; R.IVNIASVVGLVGNAGQANYSAAK.A	33383
**A9**	XP_030508280.1	Vicilin C72-like (*Cannabis sativa*)	2	1	R.ADVIVVPAGSTVYMTNQDNK.E + Oxidation (M)	99860
**A9**	XP_030493178.1	Sucrose-binding protein-like (*Cannabis sativa*)	2	2	K.LINPVSLPGR.F R.ETFNLVEGDILNIPAGTPVYIVNR.D + Deamidated (NQ)	55771
**B1**	XP_030508280.1	Vicilin C72-like (*Cannabis sativa*)	4	1	R.ADVIVVPAGSTVYMTNQDNK.E	99860
**B1**	CDP79023.1	Edestin 1 (*Cannabis sativa*)	2	2	K.TNDNAWVSPLAGR.T; R.GQGQGQSQGSQPDR.H	58810

**B3**	CDP79023.1	Edestin 1 (*Cannabis sativa*)	1	1	R.GQGQGQSQGSQPDR.H + Deamidated (NQ)	58810
**B5**	CDP79023.1	Edestin 1 (*Cannabis sativa*)	1	1	R.GQGQGQSQGSQPDR.H	58810
**B6**	CDP79023.1	Edestin 1 (*Cannabis sativa*)	9	4	R.LQGQNDDR + Deamidated (NQ);<br>K.TNDNAWVSPLAGR.T;<br>R.GQGQGQSQGSQPDR.H;<br>R.YLEEAFNVDSETVK.R + Deamidated (NQ)	58810
**B7**	XP_030508280.1	Vicilin C72-like (*Cannabis sativa*)	96	11	K.ESLQIVK ;<br>R.TVGFGVNAR.N;<br>R.ADVIVVPAGSTVYMTNQDNK.E + Oxidation (M);<br>K.FYEVTPEQNK.Q;<br>K.TTVLVMVVEGTGR.M;;<br>K.LSYFVSQQQQEEGR.G;<br>R.GHESSGPVISLQNQSPR.Y;<br>R.ADVIVVPAGSTVYMTNQDNK.E;<br>K.EAQELAFNMQGSEVEQIFNQPK.L;<br>R.VTAQLSPGDVFIIPAGHPVAVVANNNQK.L;<br>R.ADVIVVPAGSTVYMTNQDNKESLQIVK.L + Oxidation (M);	99860
**B7**	CDP79023.1	Edestin 1 (*Cannabis sativa*)	15	9	K.GTLDLVSPLR.S;<br>K.TNDNAWVSPLAGR.T;<br>R.EETVLLTSSTSSR.R;<br>R.GQGQGQSQGSQPDR.H;<br>K.QASSDGFEWVSFK.T;	58810

					R.LQGQNDDRNSIIR.V;<br>R.YLEEAFNVDSETVK.R;<br>R.YLEEAFNVDSETVK.R + Deamidated (NQ);<br>R.GILGVTFPGCPETFEESQR.G	
**B7**	XP_030493178.1	Sucrose-binding protein-like (*Cannabis sativa*)	15	5	K.ELAFSVPAR.E;<br>R.SSGPFNLFR.D;<br>K.SQNEEYFFPGPR.S;<br>R.FEPFYGAGGENPESFYK.A;<br>R.ETFNLVEGDILNIPAGTPVYIVNR.D	55771
**B7**	CDP79028.1	Edestin 2 (*Cannabis sativa*)	1	1	R.AMPDDVLANAFQISR.E + Oxidation (M)	56277
**B8**	CDP79028.1	Edestin 2 (*Cannabis sativa*)	64	12	R.GTSSPSSR.G;<br>R.GEDLQIIAPSR.I;<br>R.WQSQCQFQR.L;<br>R.SEGASSDEQHQK.V;<br>R.VRGEDLQIIAPSR.I;<br>R.AMPDDVLANAFQISR.E + Oxidation (M);<br>R.ILAESFNVDTELAHK.L;<br>R.GIHGAVIPGCPETFER.G;<br>R.ESGEQTPNGNIFSGFDTR.I;<br>R.GLLLPSFLNAPMMFYVIQGR.G + 2 Oxidation (M);<br>R.VECEAGVSEYWDIQNTEDDELHCAGVETAR.H	56277
**B8**	CDP79023.1	Edestin 1 (*Cannabis sativa*)	14	8	K.GTLDLVSPLR.S;<br>R.QQNQCQIDR.I;<br>K.TNDNAWVSPLAGR.T;<br>R.GQGQGQSQGSQPDR.H;<br>K.QASSDGFEWVSFK.T;<br>R.YLEEAFNVDSETVK.R;<br>R.FYLAGNPEDEFEQLR.R;<br>R.GILGVTFPGCPETFEESQR.G	58810

**B8**	SNQ45160.1	Edestin 3 (*Cannabis sativa*)	9	6	K.QQEGLPNNVFR.G; R.GFSVNLIQEAFNVDSETAR.K; R.TAVYGDQNECQLNR.L; R.GVLGTLFPGCAETFEEAQVSVGGGR.S; R.AMPEDVIANSYQISR.E + Oxidation (M); R.IEEEKQQEGLPNNVFR.G	56473
**B8**	XP_030493178.1	Sucrose-binding protein-like (*Cannabis sativa*)	2	1	R.ETFNLVEGDILNIPAGTPVYIVNR.D	55771
**B8**	XP_030508280.1	Vicilin C72-like (*Cannabis sativa*)	1	1	R.ADVIVVPAGSTVYMTNQDNK.E + Oxidation (M)	99860
**B9**	SNQ45160.1	Edestin 3 (*Cannabis sativa*)	97	13	K.IQSQDDFR.G; R.QELQQTER.E; R.KIQSQDDFR.G; K.QQEGLPNNVFR.G; R.TAVYGDQNECQLNR.L; R.AMPEDVIANSYQISR.E + Oxidation (M); R.FYIAGNPHEDFPQSR.R; R.IEEEKQQEGLPNNVFR.G; R.GFSVNLIQEAFNVDSETAR.K; R.VECEGGMIESWNPNHEQFQCAGVALLR.L + Deamidated (NQ); R.GVLGTLFPGCAETFEEAQVSVGGGR.S; R.LTIQPNGLHLPSYTNGPQLIHVIR.G + 2 Deamidated (NQ)	56473
**B9**	CDP79023.1	Edestin 1 (*Cannabis sativa*)	105	23	R.ADVFTPQAGR; K.GTLDLVSPLR.S; R.QQNQCQIDR.I; R.VKGTLDLVSPLR.S; K.TNDNAWVSPLAGR.T; R.EETVLLTSSTSSR.R; R.GQGQGQSQGSQPDR.H; K.QASSDGFEWVSFK.T; R.ISTVNSYNLPILR.F; R.LQGQNDDRNSIIR.V;	55810

					R.ALPEAVLANAFQISR.D; R.YLEEAFNVDSETVKR.L ; R.FYLAGNPEDEFEQLRR.E; R.RFYLAGNPEDEFEQLR.R; R.VEAEAGLIESWNPNHNQFQCAGVAVVR.Y; R.GILGVTFPGCPETFEESQR.G; R.GILGVTFPGCPETFEESQRGQGQGQSQGSQPDR.H + 2 Deamidated (NQ); R.YTIQQNGLHLPSYTNTPQLVYIVK.G + Deamidated (NQ); R.EGDIVAIPAGVAYWSYNNGDQQLVFVSLLDTSNVNNQLDDNPR.R	
**B9**	CDP79027.1	Edestin 2 (*Cannabis sativa*)	47	14	R.GEDLQIIAPSR.I; R.WQSQCQFQR.L; R.RESGEQTPNGNIFSGFDTR.I + Deamidated (NQ); R.VRGEDLQIIAPSR.I; R.LNTLNNYNLPILR.F; R.ILAESFNVDTELAHK.L; R.GIHGAVIPGCPETFER.G; R.QNIDRPSQADIFNPR.G; R.ESGEQTPNGNIFSGFDTR.I; R.GLLLPSFLNAPMMFYVIQGR.G + 2 Oxidation (M); R.VECEAGVSEYWDIQNTEDDELHCAGVETAR.H; K.EGDMVAMPAGVADWVYNNGDSPLVLIAFVDVGNQANQLDQFSR.R + 2 Oxidation (M)	56338
**B9**	XP_030506286.1	NADPH-dependent aldehyde reductase 1, chloroplastic-like (*Cannabis sativa*)	7	6	K.LLDYTATK.G; K.IALVSGGDSGIGR.A; R.VVDEVINAYGR.I; K.EGSCVINTTSVNAYK.G; R.VNGVAPGPIWTPLIPSSFSEDHTAK.F + Deamidated (NQ); R.IDILINNAAEQYVTSSIEEIDEAR.L	32167

**B9**	XP_006348126.1	Lactoylglutathione lyase GLX1 (*Solanum lycopersicum*)	4	3	K.ITSFLDPDGWK.T; K.DPDGYLFEIIQR.E; K.GNAYAQIAIGTDDVYK.S	32953
**B9**	XP_030508280.1	Vicilin C72-like (*Cannabis sativa*)	1	1	R.ADVIVVPAGSTVYMTNQDNK.E + Oxidation (M)	99860
**B9**	XP_030481474.1	Mitochondrial outer membrane protein porin of 34 kDa-like (*Cannabis sativa*)	1	1	K.SFFTISGDVDTK.A	29450
**C1**	XP_030508280.1	Vicilin C72-like (*Cannabis sativa*)	15	6	R.TVGFGVNAR.N; K.FYEVTPEQNK.Q; R.RGEQEEESQSGK.I + Deamidated (NQ); K.LSYFVSQQQQEEGR.G; R.GHESSGPVISLQNQSPR.Y; R.ADVIVVPAGSTVYMTNQDNK.E	99860
**C1**	PNY00005.1	Tubulin alpha-3 chain-like protein (*Trifolium pratense*)	5	4	R.TVQFVDWCPTGFK.C; R.AVFVDLEPTVIDEVR + Deamidated (NQ); R.AFVHWYVGEGMEEGEFSEAR.E; R.FDGAINVDITEFQTNLVPYPR.I	50123
**C1**	GAX73914.1	Hypothetical protein CEUSTIGMA_g1364.t1 (*Chlamydomonas eustigma*)	2	2	K.FWEVLSDEHGIDPTGTYHGDSDLQLER.I; K.NSSYFVEWIPNNVK.A + Deamidated (NQ)	84690
**C1**	CDP79023.1	Edestin 1 (*Cannabis sativa*)	1	1	R.GQGQGQSQGSQPDR.H	58810

**C1**	XP_030508778.1	ADP-ribosylation factor 1 (*Cannabis sativa*)	4	3	R.DAVVLVFANK.Q ; K.NISFTVWDVGGQDK.I; K.LGEIVTTIPTIGFNVETVEYK.N	20624
**C1**	XP_030493178.1	Sucrose-binding protein-like (*Cannabis sativa*)	1	1	K.SQNEEYFFPGPR.S	55771
**D1**	XP_030508280.1	Vicilin C72-like (*Cannabis sativa*)	19	7	R.TVGFGVNAR.N; K.FYEVTPEQNK.Q; K.TTVLVMVVEGTGR.M + Oxidation (M); K.LSYFVSQQQQEEGR.G; R.GHESSGPVISLQNQSPR.Y; R.ADVIVVPAGSTVYMTNQDNK.E; K.EAQELAFNMQGSEVEQIFNQPK.L + Oxidation (M)	99860
**D1**	CDP79023.1	Edestin 1 (*Cannabis sativa*)	10	7	K.GTLDLVSPLR.S; K.TNDNAWVSPLAGR.T; R.GQGQGQSQGSQPDR.H; R.LQGQNDDRNSIIR.V + 2 Deamidated (NQ); R.YLEEAFNVDSETVK.R + Deamidated (NQ); R.YLEEAFNVDSETVKR.L; R.GILGVTFPGCPETFEESQR.G	58810
**D1**	XP_030493178.1	Sucrose-binding protein-like (*Cannabis sativa*)	8	5	K.ELAFSVPAR.E; R.SSGPFNLFR.D; K.LINPVSLPGR.F; K.SQNEEYFFPGPR.S; R.FEPFYGAGGENPESFYK.A	55771
**D1**	CDP79028.1	Edestin 2 (*Cannabis sativa*)	2	2	R.GEDLQIIAPSR.I; R.AMPDDVLANAFQISR.E + Oxidation (M)	56277

**E1**	AAO45103.1	Beta-conglycinin alpha' subunit, partial (*Glycine max*)	4	4	R.LQESVIVEISK;<br>R.LQESVIVEISKeQIR.A;<br>NILEASYDTKFEEINK.V;<br>R.FESFFLSSTQAQQSYLQGFSK.N	44991
**E1**	CDP79023.1	Edestin 1 (*Cannabis sativa*)	3	3	K.TNDNAWVSPLAGR.T;<br>R.GQGQGQSQGSQPDR.H;<br>R.GILGVTFPGCPETFEESQR.G	58810
**E1**	CDP79027.1	Edestin 2 (*Cannabis sativa*)	4	2	R.GEDLQIIAPSR.I;<br>R.ESGEQTPNGNIFSGFDTR.I;	56338
**E1**	KHN10743.1	Glycinin G2 (*Glycine soja*)	2	2	K.SQSDNFEYVSFK.T;<br>R.RFYLAGNQEQEFLK.Y	59640
**E2**	SNQ45160.1	Edestin 3 (*Cannabis sativa*)	26	8	K.IQSQDDFR.G;<br>K.QQEGLPNNVFR.G;<br>R.TAVYGDQNECQLNR.L;<br>R.FYIAGNPHEDFPQSR.R;<br>R.IEEEKQQEGLPNNVFR.G;<br>R.GFSVNLIQEAFNVDSETAR.K;<br>R.GVLGTLFPGCAETFEEAQVSVGGGR.S;<br>R.VECEGGMIESWNPNHEQFQCAGVALLR.L + Oxidation (M)	56473
**E2**	CDP79028.1	edestin 2 (*Cannabis sativa*)	21	9	R.GEDLQIIAPSR.I;<br>R.WQSQCQFQR.L;<br>R.VRGEDLQIIAPSR.I;<br>R.AMPDDVLANAFQISR.E + Oxidation (M);<br>R.ILAESFNVDTELAHK.L;<br>R.GIHGAVIPGCPETFER.G;<br>R.ESGEQTPNGNIFSGFDTR.I;	56277

					R.VECEAGVSEYWDIQNTEDDELHCAGVETAR.H	
**E2**	XP_030506286.1	NADPH-dependent aldehyde reductase 1, chloroplastic-like (*Cannabis sativa*)	1	1	K.IALVSGGDSGIGR.A	32167
**E3**	CDP79023.1	edestin 1 (*Cannabis sativa*)	8	7	K.GTLDLVSPLR.S; K.TNDNAWVSPLAGR.T; R.EETVLLTSSTSSR.R; R.GQGQGQSQGSQPDR.H; K.QASSDGFEWVSFK.T; R.YLEEAFNVDSETVK.R+ Deamidated (NQ); R.GILGVTFPGCPETFEESQR.G	58810
**E3**	SNQ45153.2	7S vicilin-like protein (*Cannabis sativa*)	3	3	K.EGDVFWVPR.Y; K.SPDSYNLYDGK.K; R.ESVILPTSAASPPVK.L	56026
**E3**	CDP79028.1	edestin 2 (*Cannabis sativa*)	2	2	R.LQVVDDNGR.N; R.AMPDDVLANAFQISR.E + Oxidation (M)	56277
**F1**	CDP79023.1	Edestin 1 (*Cannabis sativa*)	7	5	K.GTLDLVSPLR.S; K.TNDNAWVSPLAGR.T + Deamidated (NQ); R.GQGQGQSQGSQPDR.H; R.YLEEAFNVDSETVK.R + Deamidated (NQ); R.GILGVTFPGCPETFEESQR.G	58810
**F1**	CDP79027.1	Edestin 2 (*Cannabis sativa*)	5	2	R.GEDLQIIAPSR.I; R.ESGEQTPNGNIFSGFDTR.I	56338
**F2**	XP_030508280.1	vicilin C72-like (*Cannabis sativa*)	73	9	K.ESLQIVK.L; R.TVGFGVNAR.N; K.FYEVTPEQNK.Q;	99860

					K.TTVLVMVVEGTGR.M;<br>K.LSYFVSQQQQEEGR.G;<br>R.GHESSGPVISLQNQSPR.Y;<br>R.ADVIVVPAGSTVYMTNQDNK.E;<br>K.EAQELAFNMQGSEVEQIFNQPK.L;<br>R.VTAQLSPGDVFIIPAGHPVAVVANNNQK.L + Deamidated (NQ)	
**F2**	CDP79023.1	Edestin 1 (*Cannabis sativa*)	32	12	R.LQGQNDDR + Deamidated (NQ);<br>R.GQGQGQSQGSQPDR.H;<br>K.GTLDLVSPLR.S;<br>R.QQNQCQIDR.I;<br>K.TNDNAWVSPLAGR.T;<br>R.EETVLLTSSTSSR.R;<br>R.GQGQGQSQGSQPDR.H;<br>R.LQGQNDDRNSIIR.V;<br>R.YLEEAFNVDSETVK.R;<br>R.YLEEAFNVDSETVKR.L;<br>R.FYLAGNPEDEFEQLRR.E;<br>R.GILGVTFPGCPETFEESQR.G	58810
**F2**	SNQ45160.1	Edestin 3 (*Cannabis sativa*)	26	5	K.QQEGLPNNVFR.G ;<br>R.TAVYGDQNECQLNR.L;<br>R.IEEEKQQEGLPNNVFR.G;<br>R.GFSVNLIQEAFNVDSETAR.K;<br>R.GVLGTLFPGCAETFEEAQVSVGGGR.S	56473
**F2**	CDP79028.1	Edestin 2 (*Cannabis sativa*)	29	8	R.GEDLQIIAPSR.I;<br>R.WQSQCQFQR.L;<br>R.RESGEQTPNGNIFSGFDTR.I;<br>R.VRGEDLQIIAPSR.I;<br>R.AMPDDVLANAFQISR.E + Oxidation (M);<br>R.ILAESFNVDTELAHK.L;<br>R.GIHGAVIPGCPETFER.G;<br>R.ESGEQTPNGNIFSGFDTR.I	56277

**F2**	XP_030493178.1	Sucrose-binding protein-like (*Cannabis sativa*)	12	5	K.ELAFSVPAR.E;<br>K.LINPVSLPGR.F;<br>K.SQNEEYFFPGPR.S;<br>R.ETFNLVEGDILNIPAGTPVYIVNR.D;<br>R.FEPFYGAGGENPESFYK.A	55771
**F2**	XP_030506286.1	NADPH-dependent aldehyde reductase 1, chloroplastic-like. (*Cannabis sativa*)	1	1	K.IALVSGGDSGIGR.A	32167
**G1**	SNQ45160.1	Edestin 3 (*C. sativa*)	41	10	K.LDLVKPQR.S;<br>R.KIQSQDDFR.G;<br>K.QQEGLPNNVFR.G;<br>R.TAVYGDQNECQLNR.L;<br>R.AMPEDVIANSYQISR.E + Oxidation (M);<br>R.FYIAGNPHEDFPQSR.R;<br>R.IEEEKEQEGLPNNVFR.G;<br>R.GFSVNLIQEAFNVDSETAR.K;<br>R.GVLGTLFPGCAETFEEAQVSVGGGR.S;<br>R.VECEGGMIESWNPNHEQFQCAGVALLR.L + Oxidation (M);	56473
**G1**	CDP79023.1	Edestin 1 (*Cannabis sativa*)	39	15	K.GTLDLVSPLR.S;<br>R.QQNQCQIDR.I;<br>R.VKGTLDLVSPLR.S;<br>K.TNDNAWVSPLAGR.T;<br>R.GQGQGQSQGSQPDR.H;<br>R.ISTVNSYNLPILR.F;<br>R.LQGQNDDRNSIIR.V;<br>R.ALPEAVLANAFQISR.D;<br>R.YLEEAFNVDSETVK.R + Deamidated (NQ);<br>R.FYLAGNPEDEFEQLR.R;<br>R.YTIQQNGLHLPSYTNTPQLVYIVK + 2 Deamidated (NQ);<br>R.FYLAGNPEDEFEQLRR.E;<br>R.VEAEAGLIESWNPNHNQFQCAGVAVVR.Y;<br>R.GILGVTFPGCPETFEESQR.G;	58810

					R.YTIQQNGLHLPSYTNTPQLVYIVK.G + Deamidated (NQ);	
**G1**	CDP79028.1	Edestin 2 (*Cannabis sativa*)	26	10	R.GEDLQIIAPSR.I; K.ASAQGFEWIAVK.T; R.VRGEDLQIIAPSR.I; R.LNTLNNYNLPILR.F; R.AMPDDVLANAFQISR.E + Oxidation (M); R.ILAESFNVDTELAHK.L; R.GIHGAVIPGCPETFER.G; R.ESGEQTPNGNIFSGFDTR.I; R.GLLLPSFLNAPMMFYVIQGR.G + 2 Oxidation (M)	56277
**G1**	SNQ45153.2	7S vicilin-like protein (*Cannabis sativa*)	4	4	K.ATEYGIILK.G; K.eGDVFWVPR.Y; R.ESVILPTSAASPPVK.L; R.AGPMEFFGFTTSAR.K + Oxidation (M)	56026
**G1**	XP_030506286.1	NADPH-dependent aldehyde reductase 1, chloroplastic-like (*Cannabis sativa*)	1	1	K.IALVSGGDSGIGR.A	32167
**H1**	CDP79023.1	Edestin 1 (*Cannabis sativa*)	2	2	K.TNDNAWVSPLAGR.T; R.GQGQGQSQGSQPDR.H	58810
**H1**	SNQ45158.1	Edestin 3 (*Cannabis sativa*)	1	1	K.EQEGLPNNVFR.G	56672
**H2**	CDP79023.1	Edestin 1 (*Cannabis sativa*)	3	3	K.TNDNAWVSPLAGR.T; R.EETVLLTSSTSSR.R; R.GQGQGQSQGSQPDR.H	58810

**H2**	SNQ45153.2	7S vicilin-like protein (*Cannabis sativa*)	3	3	K.ATEYGIILK.G; K.EGDVFWVPR.Y; K.SPDSYNLYDGK.K	56016
**H2**	CDP79027.1	Edestin 2 (*Cannabis sativa*)	1	1	R.GEDLQIIAPSR.I	56338
**I1**	SNQ45158.1	Edestin 3 (*Cannabis sativa*)	3	2	R.GFSVNLIQEAFNVDSETAR.K; R.GVLGTLFPGCAETFEEAQVSVGGGR.S	56672
**I1**	CDP79023.1	Edestin 1 (*Cannabis sativa*)	3	3	R.QQNQCQIDR.I; R.GQGQGQSQGSQPDR.H; R.GILGVTFPGCPETFEESQR.G	58810
**I2**	CDP79023.1	Edestin 1 (*Cannabis sativa*)	12	10	K.GTLDLVSPLR.S; R.QQNQCQIDR.I; K.TNDNAWVSPLAGR.T; R.GQGQGQSQGSQPDR.H; R.LQGQNDDRNSIIR.V; R.YLEEAFNVDSETVK.R; R.FYLAGNPEDEFEQLR.R; R.GILGVTFPGCPETFEESQR.G; R.YTIQQNGLHLPSYTNTPQLVYIVK + 2 Deamidated (NQ)	58810
**I2**	XP_030508280.1	vicilin C72-like (*Cannabis sativa*)	6	3	K.FYEVTPEQNK.Q; K.TTVLVMVVEGTGR.M; R.ADVIVVPAGSTVYMTNQDNK.E;	99860
**I2**	XP_030493178.1	sucrose-binding protein-like (*Cannabis sativa*)	2	2	K.ELAFSVPAR.E; K.SQNEEYFFPGPR.S	55771

**I3**	XP_030508280.1	vicilin C72-like (*Cannabis sativa*)	21	7	K.ESLQIVK; R.TVGFGVNAR.N; K.FYEVTPEQNK.Q; K.TTVLVMVVEGTGR.M; K.LSYFVSQQQQEEGR.G; R.ADVIVVPAGSTVYMTNQDNK.E; K.EAQELAFNMQGSEVEQIFNQPK.L + Oxidation (M)	99860
**I3**	CDP79023.1	Edestin 1 (*Cannabis sativa*)	8	5	K.TNDNAWVSPLAGR.T; R.GQGQGQSQGSQPDR.H; K.QASSDGFEWVSFK.T; R.YLEEAFNVDSETVK.R; R.GILGVTFPGCPETFEESQR.G	58810
**I3**	XP_030493178.1	sucrose-binding protein-like (*Cannabis sativa*)	6	3	K.ELAFSVPAR.E; K.LINPVSLPGR.F; K.SQNEEYFFPGPR.S	55771
**I3**	SNQ45160.1	Edestin 3 (*Cannabis sativa*)	2	2	K.QQEGLPNNVFR.G; K.QQEGLPNNVFR	56473
**I4**	CDP79028.1	Edestin 2 (*Cannabis sativa*)	33	10	R.LQVVDDNGR.N; R.GEDLQIIAPSR.I; R.WQSQCQFQR.L; K.ASAQGFEWIAVK.T; R.AMPDDVLANAFQISR.E + Oxidation (M); R.ILAESFNVDTELAHK.L; R.GIHGAVIPGCPETFER.G; R.ESGEQTPNGNIFSGFDTR.I; R.GLLLPSFLNAPMMFYVIQGR.G + 2 Oxidation (M)	56277

**I4**	CDP79023.1	Edestin 1 (*Cannabis sativa*)	15	9	R.ADVFTPQAGR.I;<br>K.TNDNAWVSPLAGR.T;<br>R.EETVLLTSSTSSR.R;<br>K.QASSDGFEWVSFK.T;<br>R.ISTVNSYNLPILR.F;<br>R.ALPEAVLANAFQISR.D;<br>R.YLEEAFNVDSETVK.R;<br>R.FYLAGNPEDEFEQLR.R;<br>R.GILGVTFPGCPETFEESQR.G	58810
**I4**	SNQ45160.1	Edestin 3 (*Cannabis sativa*)	6	4	K.QQEGLPNNVFR.G;<br>R.IEEEKQQEGLPNNVFR.G;<br>R.GFSVNLIQEAFNVDSETAR.K;<br>R.GVLGTLFPGCAETFEEAQVSVGGGR.S	56473
**I4**	SNQ45153.2	7S vicilin-like protein (*Cannabis sativa*)	7	4	K.ATEYGIILK.G;<br>K.EGDVFWVPR.Y;<br>K.SPDSYNLYDGK.K;<br>R.YFPFCQIASR.A	56026
**I4**	BAK03519.1	Predicted protein (*Hordeum vulgare* subs. Vulgare)	2	2	R.LIGQIVSSITASLR.F	50667
**I5**	CDP79023.1	edestin 1 (*Cannabis sativa*)	86	18	K.GTLDLVSPLR.S;<br>R.QQNQCQIDR.I;<br>R.VKGTLDLVSPLR.S;<br>K.TNDNAWVSPLAGR.T;<br>R.EETVLLTSSTSSR.R;<br>R.GQGQGQSQGSQPDR.H;<br>R.LQGQNDDRNSIIR.V + Deamidated (NQ);<br>R.ALPEAVLANAFQISR.D;<br>R.YLEEAFNVDSETVKR.L;<br>R.YLEEAFNVDSETVK.R + Deamidated (NQ);	58810

**I5**	CDP79028.1	edestin 2 (*Cannabis sativa*)	58	14	R.FYLAGNPEDEFEQLRR.E; R.VEAEAGLIESWNPNHNQFQCAGVAVVR.Y; R.GILGVTFPGCPETFEESQR.G; R.YTIQQNGLHLPSYTNTPQLVYIVK.G + Deamidated (NQ); R.LQVVDDNGR.N; R.GEDLQIIAPSR.I; R.WQSQCQFQR.L; K.ASAQGFEWIAVK.T; R.VRGEDLQIIAPSR.I; R.LNTLNNYNLPILR.F; R.AMPDDVLANAFQISR.E + Oxidation (M); R.ILAESFNVDTELAHK.L; R.GIHGAVIPGCPETFER.G; R.QNIDRPSQADIFNPR.G + Deamidated (NQ); R.RESGEQTPNGNIFSGFDTR.I; R.GLLLPSFLNAPMMFYVIQGR.G + 2 Oxidation (M); R.VECEAGVSEYWDIQNTEDDELHCAGVETAR.H	56227
**I5**	XP_004232705.1	Lactoylglutathione lyase GLX1 (*Solanum lycopersicum*)	5	3	K.ITSFLDPDGWK.T K.DPDGYLFEIIQR.E K.GNAYAQIAIGTDDVYK.S	32972
**I5**	XP_030506286.1	NADPH-dependent aldehyde reductase 1, chloroplastic-like (*Cannabis sativa*)	5	3	K.LLDYTATK.G K.IALVSGGDSGIGR.A K.EGSCVINTTSVNAYK.G	32167
**I5**	XP_030508280.1	vicilin C72-like (*Cannabis sativa*)	2	2	K.FYEVTPEQNK.Q K.LSYFVSQQQQEEGR.G	99860

**I5**	XP_030493178.1	sucrose-binding protein-like (*Cannabis sativa*)	3	3	K.ELAFSVPAR.E<br>K.LINPVSLPGR.F<br>R.FEPFYGAGGENPESFYK.A	55771
**I6**	XP_030508280.1	vicilin C72-like (*Cannabis sativa*)	29	8	K.ESLQIVK;<br>R.TVGFGVNAR.N + Deamidated (NQ);<br>K.FYEVTPEQNK.Q;<br>K.TTVLVMVVEGTGR.M;<br>K.LSYFVSQQQQEEGR.G;<br>K.EAQELAFNMQGSEVEQIFNQPK.L + Oxidation (M);<br>R.GHESSGPVISLQNQSPR.Y;<br>R.ADVIVVPAGSTVYMTNQDNK.E;	99860
**I6**	XP_030493178.1	sucrose-binding protein-like (*Cannabis sativa*)	11	4	K.ELAFSVPAR.E;<br>K.LINPVSLPGR.F;<br>K.SQNEEYFFPGPR.S;<br>R.FEPFYGAGGENPESFYK.A	55771
**I6**	CDP79023.1	edestin 1 (*Cannabis sativa*)	8	5	K.GTLDLVSPLR.S;<br>K.TNDNAWVSPLAGR.T;<br>R.GQGQGQSQGSQPDR.H;<br>K.QASSDGFEWVSFK.T;<br>R.ISTVNSYNLPILR.F + Deamidated (NQ)	58810
**I6**	SNQ45153.2	7S vicilin-like protein (*Cannabis sativa*)	5	3	K.EGDVFWVPR.Y;<br>R.ISAGSAFYiVNTGEGQR;<br>K.EILSSQQEGPIVYIPDSR.S;	56026
**I6**	CDP79028.1	edestin 2 (*Cannabis sativa*)	1	1	R.AMPDDVLANAFQISR.E	56277
**L1**	CDP79023.1	edestin 1 (*Cannabis sativa*)	18	7	R.ENIGDPSR.A;<br>R.FLQLSAER.G;<br>R.ADVFTPQAGR.I;	58810

					K.TNDNAWVSPLAGR.T;<br>R.EETVLLTSSTSSR.R;<br>K.QASSDGFEWVSFK.T;<br>R.ISTVNSYNLPILR.F;	
**L2**	CDP79023.1	edestin 1<br>(*Cannabis sativa*)	4	2	K.TNDNAWVSPLAGR.T R.EETVLLTSSTSSR.R	58810
**L2**	SNQ45158.1	edestin 3<br>(*Cannabis sativa*)	4	3	R.ADVFSPQAGR.L<br>R.CQVVDNNGR.S<br>R.AMPEDVIANSYQISR.E	56672
**L3**	SNQ45158.1	edestin 3<br>(*Cannabis sativa*)	8	4	R.ESMGDPAR.A<br>R.ADVFSPQAGR.L<br>R.QGQALTVPQNFAVVK.M<br>R.AMPEDVIANSYQISR.E	56672
**L3**	CDP79023.1	edestin 1<br>(*Cannabis sativa*)	3	1	K.TNDNAWVSPLAGR.T	58810
**L4**	CDP79028.1	edestin 2<br>(*Cannabis sativa*)	11	5	R.LQVVDDNGR.N<br>R.AMPDDVLANAFQISR.E<br>K.ASAQGFEWIAVK.T<br>K.TNDNAMRNPLAGK.V	56227
**L4**	SNQ45158.1	edestin 3<br>(*Cannabis sativa*)	4	4	R.AQVNQLAGK<br>R.ADVFSPQAGR.L<br>R.CQVVDNNGR.S<br>R.AMPEDVIANSYQISR.E	56672
**L4**	CDP79023.1	edestin 1<br>(*Cannabis sativa*)	2	1	K.TNDNAWVSPLAGR.T	58810
**L5**	CDP79023.1	edestin 1<br>(*Cannabis sativa*)	3	1	K.TNDNAWVSPLAGR.T;	58810

**L5**	CDP79028.1	edestin 2 (*Cannabis sativa*)	6	2	R.LQVVDDNGR.N + Deamidated (NQ);<br>R.AMPDDVLANAFQISR.E + Oxidation (M)	56277
**L5**	SNQ45158.1	edestin 3 (*Cannabis sativa*)	4	3	R.ADVFSPQAGR.L;<br>R.CQVVDNNGR.S + Deamidated (NQ);<br>R.AMPEDVIANSYQISR.E + Oxidation (M)	56672

Table S3. Spots with differential abundance from total protein extracts of Futura75 and Finola hemp seeds analyzed by 2D-PAGE. Spot number, ANOVA statistic parameters and samples showing changes in spot intensity are indicated.

Spot	Df	F	p-value	Differential expression
**9**	3	6,11004	<0,05	(+) Fin Vig vs Fin Cert
**12**	3	8,57694	<0,005	(+) Fin Vig
**13**	3	4,41576	<0,05	(+) Fin Cert vs Fut Cert
**22**	3	23,11334	<0,001	(+) Fin Vig
**23**	3	17,51602	<0,001	(+) Fin Vig
**33**	3	4,49596	<0,05	(+) Fin Vig vs Fut Vig
**38**	3	12,7898	<0,001	(+) Fin Vig
**39**	3	13,73631	<0,001	(+) Fin Vig
**41**	3	96,43931	<0,001	(+) Fin Vig
**42**	3	22,29541	<0,001	(+) Fin Vig, (-) Fut Vig vs Fin Vig, Fin Cert
**49**	3	81,99251	<0,001	(+) Fin Vig
**56**	3	7,99074	<0,005	(+) Fin Cert vs Fut Vig, Fin Vig
**71**	3	5,18847	<0,05	(-) Fin Vig vs Fut Vig
**72**	3	8,12546	<0,001	(+) FinCert
**73**	3	27,82664	<0,001	(-) Fin Vig, (+) Fin Cert
**74**	3	7,52056	<0,005	(-) Fin Vig vs FinCert
**75**	3	22,68718	<0,001	(-) Fin Vig
**76**	3	20,31859	<0,001	(-) Fin Vig
**80**	3	15,09476	<0,001	(+) Fin Cert, (+) Fut Cert vs Fin Vig
